# Substrates for Neuronal Cotransmission With Neuropeptides and Small Molecule Neurotransmitters in *Drosophila*

**DOI:** 10.3389/fncel.2018.00083

**Published:** 2018-03-23

**Authors:** Dick R. Nässel

**Affiliations:** Department of Zoology, Stockholm University, Stockholm, Sweden

**Keywords:** fly brain, circadian clock, olfactory system, mushroom bodies, neurosecretory cells, neuromodulation, presynaptic facilitation, short neuropeptide F

## Abstract

It has been known for more than 40 years that individual neurons can produce more than one neurotransmitter and that neuropeptides often are colocalized with small molecule neurotransmitters (SMNs). Over the years much progress has been made in understanding the functional consequences of cotransmission in the nervous system of mammals. There are also some excellent invertebrate models that have revealed roles of coexpressed neuropeptides and SMNs in increasing complexity, flexibility, and dynamics in neuronal signaling. However, for the fly *Drosophila* there are surprisingly few functional studies on cotransmission, although there is ample evidence for colocalization of neuroactive compounds in neurons of the CNS, based both on traditional techniques and novel single cell transcriptome analysis. With the hope to trigger interest in initiating cotransmission studies, this review summarizes what is known about *Drosophila* neurons and neuronal circuits where different neuropeptides and SMNs are colocalized. Coexistence of neuroactive substances has been recorded in different neuron types such as neuroendocrine cells, interneurons, sensory cells and motor neurons. Some of the circuits highlighted here are well established in the analysis of learning and memory, circadian clock networks regulating rhythmic activity and sleep, as well as neurons and neuroendocrine cells regulating olfaction, nociception, feeding, metabolic homeostasis, diuretic functions, reproduction, and developmental processes. One emerging trait is the broad role of short neuropeptide F in cotransmission and presynaptic facilitation in a number of different neuronal circuits. This review also discusses the functional relevance of coexisting peptides in the intestine. Based on recent single cell transcriptomics data, it is likely that the neuronal systems discussed in this review are just a fraction of the total set of circuits where cotransmission occurs in *Drosophila.* Thus, a systematic search for colocalized neuroactive compounds in further neurons in anatomically defined circuits is of interest for the near future.

## Introduction

Already more than 40 years ago it was proposed that individual neurons can produce more than one neurotransmitter (Burnstock, 1976), and subsequently a multitude of studies established this as a common phenomenon in central and peripheral neurons of mammals (see Hökfelt et al., 1977; Cuello, 1982; Chan-Palay and Palay, 1984; Hökfelt et al., 1987). Over the years much progress has been made in understanding the functional consequences of co-transmission in the nervous systems of vertebrates (see Svensson et al., 2001; Hnasko and Edwards, 2012; Vaaga et al., 2014). Also neurons of invertebrates, such as insects, crustaceans, and mollusks, were early on shown to co-express different neuroactive substances (O’Shea and Bishop, 1982; Adams and O’Shea, 1983; Bishop et al., 1984; Kupfermann, 1991; Weiss et al., 1992; Glantz et al., 2000; Nusbaum et al., 2001). These studies demonstrated co-expression of neuropeptides and small molecule neurotransmitters (SMNs), where the neuropeptide acts as a cotransmitter and modulates the action of the neurotransmitter (Adams and O’Shea, 1983; Blitz and Nusbaum, 1999; Glantz et al., 2000; Nusbaum et al., 2001). There were also early studies of actions of coexisting neuropeptides in neuronal circuits or at peripheral targets (Weiss et al., 1992; Nusbaum et al., 2001; Nusbaum and Blitz, 2012). From these studies, and later, it has emerged that neuropeptide cotransmission serves to increase the flexibility and the dynamic range of signaling within neuronal networks or even reconfiguring these, and thereby altering network outputs (see Marder, 2012; Nusbaum et al., 2017). It has been shown that neuropeptides can diffuse some distance within the CNS and that they can act extrasynaptically, even far from the release site [(Jan and Jan, 1982) and reviewed in (Zupanc, 1996; Nässel, 2009; Nusbaum and Blitz, 2012; van den Pol, 2012)]. This increases the flexibility of neuromodulation with action both near the synapse and away from it. Although not yet demonstrated in insects, it is known from mammals that also SMNs (e.g., monoamines and amino acids) can diffuse some distance from the synapse (synaptic spillover or parasynaptic signaling) and act on receptors away from the nearest target neuron (Agnati et al., 1995; Szapiro and Barbour, 2009). Still, it appears as if the fast SMNs are more confined to the hardwiring of the synapses, whereas the neuropeptides have freedom to act at other sites, by so-called volume transmission (Agnati et al., 1995). Another difference is the temporal scale of action with SMNs operating in a millisecond range and neuropeptides commonly over seconds, minutes or longer, and the neuropeptides may require a stronger stimulus (stronger depolarization or burst of action potentials) to be released (Merighi, 2002; Marder, 2012; van den Pol, 2012; Nusbaum et al., 2017). Some neuropeptides are only released episodically and in bulk, especially ones constituting developmental signals (see Kim et al., 2006; Park et al., 2008). It is also known that neuropeptides can act in autocrine loops to regulate release of SMNs from the same neuron by presynaptic facilitation (Merighi, 2002; Root et al., 2011) or act in retrograde feedback to input neurons (Hu et al., 2017). Furthermore, colocalized neuropeptides can engage in various forms of intrinsic and extrinsic neuromodulation, where the peptide can be released by neurons within the circuit or from ones extrinsic to it (Katz and Frost, 1996; Katz, 1998; Morgan et al., 2000; Marder and Bucher, 2007; Nusbaum and Blitz, 2012).

The repertoire of possible actions of colocalized substances in insects has been extended substantially with the discovery that also neurosecretory cells and gut endocrine cells can produce multiple peptide hormones that may act both locally in paracrine signaling, at peripheral targets and in certain neuronal circuits in the CNS to orchestrate physiology and behavior (Veenstra et al., 2008; Kahsai et al., 2010; Nässel and Winther, 2010; Veenstra and Ida, 2014; Wegener and Veenstra, 2015; Zandawala et al., 2018). More recently, studies employing single cell transcriptomics have expanded the list of colocalized neuroactive substances in *Drosophila* neurons (Abruzzi et al., 2017; Croset et al., 2017; Davie et al., 2017).

This review presents a summary of neurons and other cells in *Drosophila* that employ two or more colocalized peptides, or peptides coexisting with SMNs, including monoamines, amino acids and acetylcholine. Such coexistence occurs in neurosecretory cells, interneurons, sensory cells and motor neurons, as well as in endocrine cells of the intestine, indicating that neuropeptides act as local or more global neuromodulators, circulating hormones and hormone releasing factors or as cotransmitters of SMNs. Emphasis is on neuromodulation in clock circuits, olfactory and mechanosensory systems, mushroom bodies (MBs) and the neuromuscular junction, as well as hormone actions of peptides coreleased from neurosecretory/endocrine cells of the brain, ventral nerve cord (VNC) and intestine.

**FIGURE 1:**
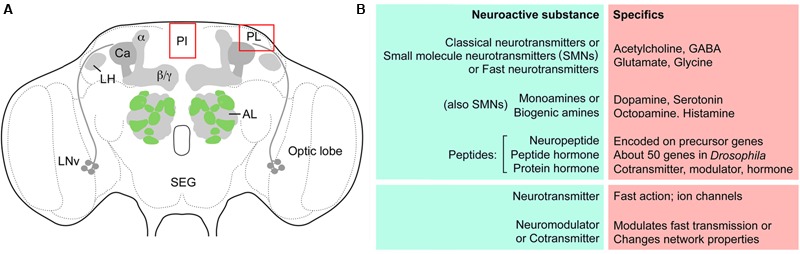
Outline of *Drosophila* brain centers and some definitions. **(A)** The adult *Drosophila* brain with some of the centers/regions discussed in this review. The glomerular antennal lobes (AL) are located anteriorly. Projection neurons from the ALs supply axons to the calyx (Ca) of the mushroom bodies (MBs) and the lateral horn (LH). The MBs have three major lobes, α, β, and γ lobes. Neurosecretory cells are located in the pars intercerebralis (PI) and pars lateralis (PL), as well as in the subesophageal ganglion (SEG). Bilateral pacemaker centers of the circadian clock are located near the optic lobe and are formed by the LNv clock neurons and others not shown. **(B)** Some definitions of terms used in this review for signaling with neuroactive substances. So-called classical- or SMNs are sometimes referred to as fast neurotransmitters. The four listed ones are discussed in this review. Monoamines or biogenic amines can be subdivided into catecholamines, indolamines and phenolamines, and the four amines discussed here are listed. Neuropeptides/peptide hormones are very diverse and some larger ones are termed protein hormones. Commonly peptides/proteins of this kind act as cotransmitters, modulators or hormones over a slower and more long lasting temporal scale than the neurotransmitters. The neurotransmitters often activate ion channels leading to rapid changes in membrane potential.

Since the terminology is somewhat diverse in different descriptions of neuroactive substances and their actions, some definitions and synonyms are provided in **Figure 1**. This figure also shows a schematic of the *Drosophila* brain with some of the structures discussed in this review. Abbreviations used in this review are listed in **Table 1**.

**Table 1:** Abbreviations.

General abbreviations	Neuropeptide/neurotransmitter acronyms
ABLKs, abdominal leucokinin producing neurons	Ach, acetylcholine AKH, adipokinetic hormone
CA, corpora allata	AstA, allatostatin A
CC, corpora cardiaca	AstC, allatostatin C
Cha, choline acetyltransferase dInR, *Drosophila* insulin receptor	Capa-PK, capa-pyrokinin (from *capability* gene)
DLPs, dorso-lateral peptidergic neurons (Crz expressing)	CapaPVK, capa-periviscerokinin (from *capability* gene)
DN, dorsal neurons (clock neurons, DN1-3)	CCAP, crustacean cardioactive peptide Crz, corazonin
DP1, dorsal paired neurons (1st abdominal ganglion)	DH31 and DH44, diuretic hormones 31 and 44
EECs, enteroendocrine cells (gut endocrines)	DILP1-3, 5 and 7, *Drosophila* insulin-like peptides 1-3, 5 and 7
GAD-1, glutamic acid decarboxylase-1	DSK, drosulfakinin
IPCs, insulin-producing cells (in brain) LNs, local neurons (interneurons in antennal lobe)	hug-PK, hugin-pyrokinin (from *hugin* gene) LK, leucokinin
LNCs, lateral neurosecretory cells LN_v_ (l-LN_v_, s-LN_v_), lateral neurons, ventral (large and small) LN_d_, lateral neurons, dorsal MB, mushroom body MBON, mushroom body output neuron	MIP, myoinhibitory peptide (aka Allatostatin B) NPF, neuropeptide F PDF, pigment-dispersing factor sNPF, short neuropeptide F TK, tachykinin (aka DTK)
MNC, median neurosecretory cell	
OSN, olfactory sensory neuron	
PN, projection neuron (antennal lobe)	
SEG, subesophageal ganglion (aka subesophageal zone)	
SMN, small molecule neurotransmitter	
Upd1, unpaired-1(leptin-like cytokine)	
vAChT, vesicular acetylcholine transporter	
vGluT, vesicular glutamate transporter	
VNC, ventral nerve cord	

## Neuroactive Compounds that Have Been Found Colocalized in Neurons of *Drosophila*

### Colocalization of Neuropeptides and Small Molecule Neurotransmitters

Out of all the neuropeptides and peptide hormones encoded by more than 50 genes known in *Drosophila* (see Nässel and Winther, 2010; Ida et al., 2011a,b; Jiang et al., 2013; Jung et al., 2014; Yeoh et al., 2017), very few have so far been mapped to neurons that coexpress other peptides or SMNs. This is primarily due to the fact that no systematic analysis of colocalization has been attempted. The following neuropeptides are known to be expressed in specific *Drosophila* neurons and endocrine cells that also utilize other neuroactive compounds based on immunocytochemistry, Gal4 expression or other “mapping” techniques (**Table 2**): adipokinetic hormone (AKH), allatostatin C (AstC), bursicon, Capa-pyrokinin/periviscerokinin (Capa-PK/PVK), crustacean cardioactive peptide (CCAP), corazonin (Crz), diuretic hormones 31 and 44 (DH31 and DH44), *Drosophila* insulin-like peptides 1–3, 5, and 7 (DILP1-3, 5 and 7), drosulfakinin (DSK), hugin-pyrokinin (hug-PK), IPNamide, leucokinin (LK), limostatin, myoinhibitory peptide (MIP), neuropeptide F (NPF), orcokinin B, partner of bursicon, pigment-dispersing factor (PDF), proctolin, short neuropeptide F (sNPF), and tachykinin (TK). References to these studies are provided in **Table 2**. There are also a few SMNs that have been shown to coexist with neuropeptides in *Drosophila* (**Table 2**). These are acetylcholine, GABA, glutamate and glycine and are commonly detected by indirect means. Presence of acetylcholine is in most cases based on antisera to choline acetyltransferase (Cha) and vesicular acetylcholine transporter (vAChT), or on *Cha* promoter Gal4-driven GFP expression (Yasuyama et al., 1995; Salvaterra and Kitamoto, 2001; Kolodziejczyk et al., 2008; Barnstedt et al., 2016). GABA detection relies on glutamic acid decarboxylase-1 (GAD-1) Gal4 expression and GABA or GAD-1 immunolabeling (Enell et al., 2007; Kolodziejczyk et al., 2008). Glutamate localization is indicated by immunolabeling with antisera to glutamate, vesicular glutamate transporter (vGluT) and vGluT-Gal4 expression (Mahr and Aberle, 2006; Hamasaka et al., 2007; Daniels et al., 2008; Kolodziejczyk et al., 2008).

**Table 2:** Colocalization of neuropeptides with neuropeptides and other neuroactive substances in neurons and endocrine cells of *Drosophila* established by marker techniques^1^.

Tissue^2^	Cell type^2^	Substances^3^	Reference
Brain	IPCs (NSCs; PI)	DILP1, 2, 3, 5, DSK	Brogiolo et al., 2001; Söderberg et al., 2012; Liu et al., 2016a
Brain	MNCs (NSCs; PI)	DH44, DILP2	Ohhara et al., 2018
Brain	DLP (NSCs; PL)	CRZ, sNPF, proctolin	Isaac et al., 2004; Kapan et al., 2012
Brain	ipc-1 (NSCs; PL)	ITP, sNPF, TK	Kahsai et al., 2010
Brain	l-LNv (clock neurons)	PDF, NPF, Upd1	Schlichting et al., 2016; Beshel et al., 2017
Brain	s-LNv (clock neurons)	PDF, sNPF, glycine^4^	Johard et al., 2009; Frenkel et al., 2017
Brain	5th s-LNv (clock neurons)	ITP, NPF, Ach^4^	Johard et al., 2009; Schlichting et al., 2016
Brain	LNd (clock neurons)	ITP, NPF	Johard et al., 2009
Brain	LNd (clock neurons)	sNPF, Ach^4^	Johard et al., 2009
Brain	DN1a (clock neurons)	DH31, IPNamide, Glutamate^4^	Shafer et al., 2006; Hamasaka et al., 2007; Goda et al., 2016
Brain	DN1p (clock neurons)	DH31, Glutamate^4,5^	Hamasaka et al., 2007; Kunst et al., 2014
Brain	LN (local neurons; AL)	MIP, Ach^4^	Carlsson et al., 2010
Brain	LN (local neurons; AL)	AstA, Ach^4^	Carlsson et al., 2010
Brain	LN (local neurons; AL)	TK, GABA^4^	Ignell et al., 2009
Brain	LN (local neurons; AL)	TK, MIP	Carlsson et al., 2010
Brain	LN (local neurons; AL)	TK, Ast-A	Carlsson et al., 2010
Brain	LN (local neurons; AL)	MIP, Ast-A	Carlsson et al., 2010
Brain	OSNs (sensory; AL)	sNPF, Ach^4^	Buchner et al., 1986; Nässel et al., 2008
Brain	OSNs (sensory; AL)^6^	MIP, Ach^4^	Hussain et al., 2016a
Brain	Kenyon cells (MB)	sNPF, Ach^4^	Johard et al., 2008; Barnstedt et al., 2016
Brain	NPF interneurons^7^	NPF, sNPF	Nässel et al., 2008
Brain	Small interneurons	sNPF, GABA^4^	Nässel et al., 2008
Brain	Small interneurons	sNPF, Ach^4^	Nässel et al., 2008
Brain	Small interneurons	sNPF, glutamate^4^	Nässel et al., 2008

SEG	Hugin neurons (L1)^8^	Hug-PK, Ach^4^	Schlegel et al., 2016
SEG	Large SEG neurons	Capa-PK, Hug-PK_2-15_	Wegener et al., 2006

CC	Corpora cardiaca cells	AKH, Limostatin	Lee and Park, 2004; Alfa et al., 2015

VNC	ABLK (NSCs)	LK, DH44	Zandawala et al., 2018
VNC	DP1 (interneurons; L3)	DILP7, sNPF, Ach^4^	Nässel et al., 2008; Hu et al., 2017
VNC	CCAPa (NSCs; L3)	CCAP, Bursicon	Luan et al., 2006
VNC	CCAPp (NSCs; L3)	CCAP, Bursicon, MIP	Kim et al., 2006
VNC	Motoneurons (RP2; L3)	Proctolin, glutamate^4^	Luo et al., 2017
VNC	CRZ neurons (males)	CRZ, Ach^4^	Tayler et al., 2012

Midgut	Endocrine cells	TK, NPF	Veenstra et al., 2008
Midgut	Endocrine cells, posterior	TK, DH31	Veenstra et al., 2008
Midgut	Endocrine cells, middle	Ast-C, Orcokinin B	Veenstra and Ida, 2014
Midgut	Endocrine cells, L3	MIP, Ach^4^	LaJeunesse et al., 2010

*^*1*^In adults, unless otherwise specified (L1 and L3, 1st and 3rd instar larvae). ^*2*^Abbreviations (some acronyms are established names of neurons, and not explained here): SEG, subesophageal ganglion; CC, corpora cardiaca; VNC, ventral nerve cord; IPCs, insulin producing cells; NSCs, neurosecretory cells; PI, pars intercerebralis; PL, pars lateralis; AL, antennal lobe; OSNs, olfactory sensory neurons (antennae to brain); MB, mushroom body. ^*3*^Abbreviations of peptides/transmitters as in text and in **Table 1**. If not otherwise specified (details in text) determined by immunocytochemistry and/or Gal4 expression (details in text); in some cases antisera to biosynthetic enzymes. ^*4*^Detected by promoter-Gal4 expression or antisera to biosynthetic enzymes ^*5*^Note that DN1p constitute a cluster of neurons and individual ones have not been investigated; these cells appear heterogeneous in terms of transmitters/modulators (see also **Figure 5E**). ^*6*^In female flies. ^*7*^Two dorsal abdominal neurons shown in **Figure 3B**. ^*8*^In hugin-PC and hugin-VNC/PH cells.*

Surprisingly, there is only one report on mapping a neuropeptide together with a biogenic amine in specific neurons of *Drosophila* (Castellanos et al., 2013), although this has been demonstrated more frequently in other insects such as moths, locusts and cockroaches and especially in mammals [summarized in (Hökfelt et al., 1987; Nässel, 2002; Nässel and Homberg, 2006)]. In the lamina of the *Drosophila* visual system there are cases of colocalized SMNs such as GABA and acetylcholine in C2 neurons, as well as acetylcholine and glutamate in L1 and L2 neurons (see Kolodziejczyk et al., 2008).

The studies discussed above are based on traditional imaging techniques and have probably only revealed the tip of the iceberg. Recently, reports on single-cell transcriptomics of dissociated *Drosophila* brain neurons discovered numerous additional patterns of colocalized neuropeptides and neuropeptides with SMNs (Abruzzi et al., 2017; Croset et al., 2017; Davie et al., 2017), shown in **Tables 3, 4**. Although this type of analysis substantially increased the cases of likely colocalization of neuropeptides and monoamines, as well as other SMNs, it provides little information on which specific types of neurons that express the substances. Therefore it is urgent to localize the proposed SMNs and neuropeptides to specific brain neurons *in situ* using conventional mapping techniques to allow for circuit analysis. Such charting is likely to unveil a huge complexity in cotransmission in neuronal networks in the *Drosophila* brain. The examples of colocalized neuropeptides given above pertain to peptides that arise from distinct precursor genes. In the next section I will discuss coexpression of multiple neuropeptides derived from the same precursor gene.

**Table 3:** Neuropeptide transcripts expressed in *Drosophila* brain neurons sorted by neurotransmitter phenotype as determined by single cell transcriptomics (Davie et al., 2017).

	Peptide										
**Monoamine**	
Serotonin	DILP7	NPLP1	MIP	DH44	proct	NPLP2					
Dopamine				DH44			CCH2a	FMRFa			
Octopamine			MIP		Proct						DH31
**Transmitter**	
Cholinergic	DILP7						CCHa2	FMRFa	CCHa1	Capa	NPLP3
GABA		NPLP1						FMRFa			
Glutamate		NPLP1	MIP	DH44	Proct		CCHa2	FMRFa	CCHa1		

*RNA sequence data from dissociated neurons were clustered to neuron types according to expression of specific neurotransmitter markers, such as vAChT/ChAT, vGluT, Gad1, SerT/Trh, Tdc/Vmat, and Ple/Vmat. For peptide acronyms see text and **Table 1**. Gray lettering denotes weak expression. Data mined from Davie et al. (2017).*

**Table 4:** Colocalized neuropeptides, monoamines, and neurotransmitters in *Drosophila* brain neurons based on single cell transcriptomics (Croset et al., 2017).

	Peptide										
**Monoamine**											
Dopamine	DH44	NPLP1	Gpb5	Proct							
Serotonin	**DH44**	NPLP1	Gpb5						sNPF		
Octopamine	DH44	**NPLP1**	Gpb5		SIFa	ITP	**DMS**	CAPA			
Tyramine		NPLP1	Gpb5		**SIFa**	ITP			sNPF	**MIP**	**DH31**
**Transmitter**											
Cholinergic	sNPF	CCHa2	TK								
GABA	DH31										
Glutamate	NPLP1	AstA									
2 or 3 of the above	DMS										

*RNA sequence data from dissociated neurons were clustered to neuron types according to expression of specific neurotransmitter markers as in **Table 3**. Bold text denotes strong expression. For peptide acronyms see text and **Table 1**. Gpb5, glycoprotein B5. From Croset et al. (2017).*

### Colocalization of Neuropeptides Derived From the Same Precursor Gene

Neuropeptide colocalization can also arise from expression of genes encoding precursors that can produce multiple copies of sequence-related peptide isoforms, or in a few cases precursors that generate peptides that might be functionally distinct. One example of the former is the thoracic Tv1-3 neurons that produce 8 different extended FMRFamide-like peptides derived from the same gene. Five of these peptides have variable N-terminal sequences, but a conserved FMRFamide C-terminus; the sequences of the other peptides differ more overall (Schneider and Taghert, 1988; Wegener et al., 2006). It appears that seven of these FMRFamides are functionally redundant in modulating the nerve-stimulated contraction of larval body wall muscles (Hewes et al., 1998). Several other *Drosophila* prepropeptides (peptide precursors) can give rise to multiple neuropeptides with related sequences (e.g., Ast-A, MIP, natalisin, sNPF, and TK), but so far there are no studies that suggest distinct differential functions (only slightly different potencies) of these sequence-related peptide isoforms in *Drosophila* or other insects (see Lange et al., 1995; Nässel and Winther, 2010; Jiang et al., 2013). It may be relevant to mention here that although isoform multiplication within a precursor may result in a diversification of functional neuropeptides over evolution, analysis of the genomes of 12 *Drosophila* species revealed a remarkable conservation of isoform sequences between these species (Wegener and Gorbashov, 2008). This suggests that peptides with biological activity are under stabilizing selection, but certainly some isoform diversification has occurred in some precursor genes during earlier evolution.

Some evidence for differential actions of sequence-related peptide isoforms exists in other animals. In mollusks a few sets of peptide isoforms derived from single precursors have been tested on different muscle preparations and these studies revealed that, depending on the dose, some isoforms can produce differential modulatory actions on contractions, whereas others are redundant (Brezina et al., 1995; van Golen et al., 1996). A more striking example of differential actions of sequence-related isoforms derived from a single precursor is provided by mammalian preprotachykinin A that gives rise to substance P and neurokinin A, which have different affinities for the three receptors NK1-3 that have different distributions (see Otsuka and Yoshioka, 1993).

The second variety of peptide precursors which generates colocalized peptides with distinct sequences can be exemplified by the one encoded on the *Capa* gene in *Drosophila.* This produces two distinct types of peptides Capa-PK and Capa-PVK1 and 2 (Capa1 and 2) (Kean et al., 2002). However, only the actions of the PVKs have been studied so far and the Capa-PK function remains to be determined. An excellent example of functional roles of distinct neuropeptides generated from the same precursor is from the snail *Lymnaea* where the FMRFamide gene encodes multiple peptides, several of which have sequences distinct from FMRFamide (Santama et al., 1995; Santama and Benjamin, 2000). Due to differential splicing of this gene, sets of distinct peptides (tetrapeptides and heptapeptides) are targeted to two specific neuron populations in a mutually exclusive pattern and serve in distinct aspects of heart regulation (Santama and Benjamin, 2000). In *Drosophila* the *Itp* gene is known to produce three splice forms, each giving rise to a distinct peptide (Dircksen et al., 2008). The resulting peptides, ITP, ITPL1, and ITPL2 differ somewhat in their sequences and only ITP is C-terminally amidated; these peptides are likely to display differential expression patterns and maybe distinct functions (Dircksen et al., 2008), but details are yet to be revealed.

Further discussion of functions of neuropeptides encoded by the same gene, both redundancy and differential roles can be found in (Nässel, 1996; Santama and Benjamin, 2000; Wegener and Gorbashov, 2008). As noted, the functional aspects of coexpressed neuropeptide isoforms is underexplored in *Drosophila* and certainly would merit a more systematic exploration in the future, especially for peptides derived from genes encoding Capa, NPLP1, and sNPF.

In the following sections I will discuss specific cases of colocalized substances in *Drosophila* neurons and neuronal circuits, as well as neuroendocrine cells in the CNS and elsewhere, including the intestine. When possible the functional implications of co-expressed substances will be discussed. However, I will not further deal with coexpression of neuropeptides derived from the same precursor gene.

## Colocalized Peptides in Neuroendocrine Cells in the Brain and Ventral Nerve Cord

This section is a survey of coexpression of neuroactive compounds in neurosecretory and neuroendocrine cells of the brain and VNC, as well as in motor neurons and other efferent neurons. Neurosecretory cells release peptide hormones into the circulation to target a host of different tissues to regulate for instance metabolic homeostasis, diuresis, reproduction and developmental transitions (see Nässel and Winther, 2010). Peptide hormones are commonly released episodically and act over extended periods. Co-expressed peptide hormones may after release act on different or overlapping targets to orchestrate organismal responses (Zandawala et al., 2018). Since neurosecretory cells have processes also within the CNS that express peptides it is not unlikely that they act within central circuits or interact with other neurosecretory cells (Kapan et al., 2012). Although not explicitly tested in insects, it might be that a neuropeptide colocalized with a typical peptide hormone acts within the CNS only, or in an autocrine regulation of hormone release at the axon termination. In the following cases of coexpressed peptide hormones are discussed, but also neuropeptides coexpressed in interneurons and efferent neurons innervating muscle fibers and reproductive organs.

### Coexpression in Neurosecretory Cells in the Brain and Roles in Hormonal Signaling

**FIGURE 2:**
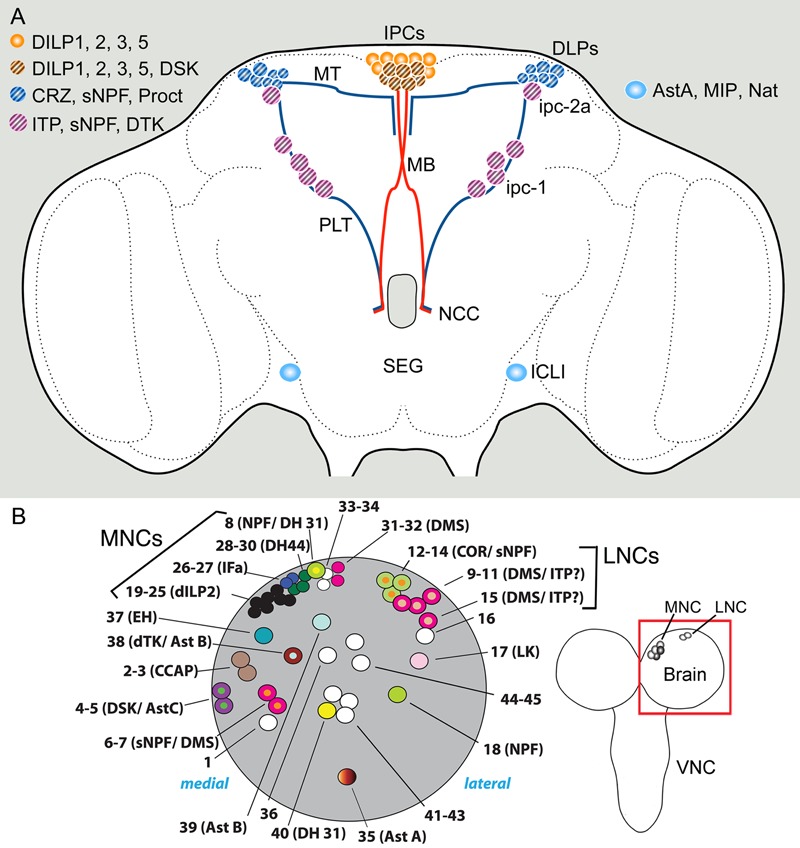
Sets of neuroendocrine cells in the *Drosophila* brain that express colocalized neuropeptides and peptide hormones. **(A)** Neurosecretory cells and interneurons of the adult brain. The insulin producing cells (IPCs) co-express DILP1, 2, 3, and 5. Some of the IPCs (cross hatched) also produce drosulfakinins (DSK1 and 2). The IPCs are regulated by DLP neurons that produce short neuropeptide F (sNPF), corazonin (CRZ), and proctolin (Proct) (Kapan et al., 2012). These DLPs are likely to also release CRZ into the circulation via axons passing through the NCC nerves to peripheral release sites in the CC, anterior aorta and intestine (Kubrak et al., 2016). Another set of lateral neurosecretory cells (LNCs), designated ipc-1 and ipc-2a, express the peptides sNPF, tachykinin (TK) and ion transport peptide (ITP) (Kahsai et al., 2010). These neurons, like the DLPs, are parts of the LNC clusters and have axon terminations in peripheral sites overlapping those of the IPCs and the DLPs. A pair of large interneurons in the brain (ICLI) were shown to co-express AstA, MIP and natalisin Diesner et al. (2018). Further abbreviations: MT, medially projecting axon tract; PLT, posterior lateral axon tract; MB, median bundle. This figure is slightly altered and updated from Nässel et al. (2013). **(B)** Neuroendocrine cells in one hemisphere of the larval brain (dorsal view). The cells shown (numbered 1–43) are Dimmed positive cells, most of which have neuropeptides assigned to them. The *bona fide* MNCs include cells numbered 19–25, 28–30, and 31–32, the other adjacent neurons are peptidergic interneurons. Note several cases of colocalized peptides, some of which so far have not been observed in the adult brain: NPF/DH31, DMS/ITP, dTK/AstB, DSK/AstC, and sNPF/DMS (acronyms as in text). Left image from Park et al. (2008) with permission (PLOS, Open access).

In the brain of insects there are two major sets of neurosecretory cells, median neurosecretory cells (MNCs) and lateral neurosecretory cells (LNCs), both located dorsally in the protocerebrum (see Siegmund and Korge, 2001; Hartenstein, 2006) (**Figure 2A**). There are some additional neurosecretory cells in the subesophageal ganglion (SEG). In *Drosophila* the brain neurosecretory cells send axons to peripheral release sites (neurohemal areas) in the corpora cardiaca (CC) and/or corpora allata (CA) located at the junction between foregut and proventriculus anteriorly in the thorax, as well as the surface of the aorta, anterior foregut, proventriculus, and crop, thereby providing extensive release sites in contact with the open circulation (Siegmund and Korge, 2001; Hartenstein, 2006).

Peptides expressed in different sets of MNCs are *Drosophila* insulin-like peptides (DILPs) DILP1, 2, 3, and 5, sulfakinin (DSK), diuretic hormone 44 (DH44), and dromyosuppressin (DMS) (see Park et al., 2008; Nässel and Vanden Broeck, 2016) (**Figure 2A**). Probably more peptides are present in other MNCs, such as allatostatin B (AstB, also known as myoinhibitory peptide, MIP) and allatostatin C (AstC) (Williamson et al., 2001a,b; Min et al., 2016). The different LNCs express allatostatin A (AstA), corazonin (Crz), sNPF, proctolin, ion transport peptide (ITP), prothoracicotropic hormone (PTTH), and tachykinin (TK, also known as DTK) (Yoon and Stay, 1995; Mc Brayer et al., 2007; Kahsai et al., 2010; Kapan et al., 2012).

In some of the neurosecretory systems listed above colocalized peptides have been detected (**Figure 2A**). The following description pertains to adult *Drosophila* if not elsewise specified. The insulin-producing cells (IPCs) of the MNC group are known to coexpress DILP1, 2, 3, and 5, which are each encoded by a separate gene (Brogiolo et al., 2001; Rulifson et al., 2002; Liu et al., 2016a). DILP1 is only expressed transiently during pupal stages and the first few days of adult life, whereas the other DILPs are expressed throughout larval, pupal, and adult stages (see Liu et al., 2016a). Several studies have suggested that the different DILPs are regulated individually at the transcriptional level (summarized in Ikeya et al., 2002; Grönke et al., 2010; Nässel et al., 2015; Nässel and Vanden Broeck, 2016) and also that release of the peptides from the IPCs is likely controlled separately for each DILP (Geminard et al., 2009; Kim and Neufeld, 2015). This requires that the different DILPs are located in different vesicle populations, which was proposed for DILP2 and 3 based on immunolabeling (Kim and Neufeld, 2015). There is also evidence that a multitude of different factors trigger transcriptional activation or release of the different DILPs in different combinations, further supporting that each of the colocalized peptide hormones is regulated separately [summarized in (Nässel et al., 2013; Alfa and Kim, 2016; Nässel and Vanden Broeck, 2016)]. These factors include nutrients, SMNs, neuropeptides and fat body derived factors. Taken together it is suggestive that the four DILPs colocalized in the IPCs have distinct functions during development and in the daily life of *Drosophila*, although some redundancy between the peptides has been demonstrated (Grönke et al., 2010). These DILP functions include regulation of growth, carbohydrate and lipid metabolism and storage, stress responses, fecundity and lifespan (Brogiolo et al., 2001; Tatar et al., 2001, 2014; Rulifson et al., 2002; Grönke et al., 2010; Alfa and Kim, 2016).

In addition to the DILPs, the IPCs also produce drosulfakinins (DSK1 and 2), two cholecystokinin-like peptides (Park et al., 2008; Söderberg et al., 2012). Although DSK can be found in additional brain neurons, DSK in the IPCs seems sufficient to induce satiety in flies (Söderberg et al., 2012). Since DILPs (at least certain ones) are released after feeding, and can induce satiety, the colocalized DSK may act together with DILPs to orchestrate post-feeding physiology. In *Drosophila* DSK is also known to regulate gut function, hyperactivity and aggression (summarized in Nässel and Williams, 2014; Williams et al., 2014).

**FIGURE 3:**
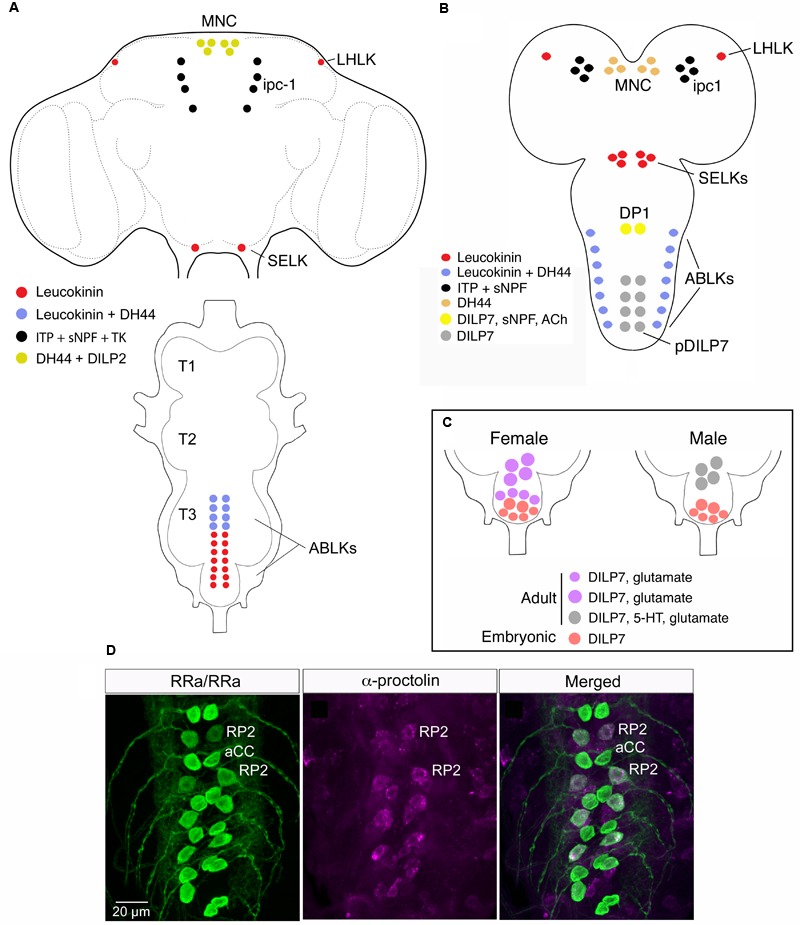
Neuroendocrine cells colocalizing neuropeptides and neurotransmitters in the CNS of adult and larval *Drosophila*. **(A)** Neuroendocrine cells colocalizing leucokinin and diuretic hormone 44 in the adult CNS. In the brain there are 4 neurons expressing leucokinin (LK), 6 neurons producing diuretic hormone 44 (DH44) and 8 ipc-1 neurons producing ion transport peptide (ITP; shown because they express Lk-Gal4), tachykinin (TK) and sNPF. In the abdominal ganglion at least 8 of the 22 ABLKs coexpress LK and DH44. Note that in adults, but not larvae the brain MNCs coexpress DH44 and DILP2 (Ohhara et al., 2018). This figure is slightly altered from Zandawala et al. (2018). **(B)** In third instar larvae all ABLKs coexpress LK and DH44. In younger larvae the ipc-1 neurons, in addition to ITP and sNPF, express weak LK (de Haro et al., 2010; Kahsai et al., 2010). One subset of midline neurons expressing DILP7, the DP1 neurons, coexpress sNPF and acetylcholine (Ach) (Nässel et al., 2008). **(C)** In adult flies the posterior DILP7 expressing neurons in abdominal ganglia consist of neurons derived from the embryo (pDILP7 in **B**) and adult-specific ones that are generated later. There is a sex-dimorphism in that females have a larger number of adult DILP7 neurons that also produce glutamate, whereas in males the adult DILP7 neurons produce additional serotonin (5-HT) (Castellanos et al., 2013). **(D)** In each neuromere of the larval ventral nerve cord (VNC) there is a pair of midline motor neurons, aCC and RP2 that signal with glutamate. The RP2 neurons also produce proctolin from Luo et al. (2017).

Recently it was shown that in adult flies the six DH44 producing MNCs also produce weak DILP2 expression (Ohhara et al., 2018) (see **Figures 3A, 7A** for DH44 neurons). Thus, there are possibly novel roles of DILP2 associated with activation of the DH44 neurons.

Among the LNCs there is a set of neurons, designated DLPs, that produces Crz, sNPF and proctolin (Isaac et al., 2004; Nässel et al., 2008; Kapan et al., 2012) (**Figure 2A**). These neurons use sNPF to regulate IPCs, and Crz for systemic signaling to regulate metabolism and stress responses (Kapan et al., 2012; Kubrak et al., 2016). Both sNPF and Crz have been extensively studied in other contexts in *Drosophila*. Accumulated data suggest that in *Drosophila* Crz is involved in stress signaling, metabolism, sperm transfer, and copulation (see Veenstra, 2009; Zhao et al., 2010; Tayler et al., 2012; Kubrak et al., 2016) whereas sNPF is truly multifunctional, including roles as a pre- and postsynaptic cotransmitter [summarized in (Nässel and Wegener, 2011; Hu et al., 2017) and discussed in later sections]. Since there is no evidence for hormonal functions of sNPF it is possible that its role in DLP neurons is confined to the brain, such as being a regulator of IPCs, or even as a regulator or hormone release in the CC. The role of proctolin in DLPs has not been investigated, although in locusts proctolin (probably from similar LNCs) has been shown to trigger release of AKH from the CC and stimulate juvenile hormone biosynthesis in the CA (Clark et al., 2006).

Another set of prominent neurosecretory cells of the brain is the 10 ITP producing ipc-1 and Ipc-2a neurons with cell bodies in the LNC group and axons to the CC/CA, aorta and anterior intestine (Dircksen et al., 2008) (**Figures 2A, 3A**). These cells were found to colocalize ITP, sNPF, and tachykinin (TK) in adult flies (Kahsai et al., 2010), and maybe in addition leucokinin (LK) at least in early larvae (Herrero et al., 2007). Knockdown of either sNPF or TK in ipc-1/Ipc-2a neurons leads to decreased survival during desiccation and starvation, as well as an increase in water loss during desiccation (Kahsai et al., 2010). The role of ITP was not studied at the time due to lethality associated with its non-conditional knockdown. It is possible that ITP acts in *Drosophila* as an antidiuretic hormone, since in the locust ITP was shown to stimulate Cl^-^ transport from gut lumen to the circulation and thereby reabsorb water (Audsley et al., 1992; Coast et al., 2002). Thus, the ipc-1/Ipc-2a neurons appear to utilize three different neuropeptides to control water homeostasis and responses associated with metabolic and ionic stress. While these neurons supply axon terminations also to the CA, it is possible that one or several of the peptides are involved in regulating juvenile hormone biosynthesis, together with insulins from the IPCs (Tu et al., 2005). In the flour beetle *Tribolium castaneum* knockdown of ITP induced reduced fecundity and in larvae ITP is important in molting behavior (Begum et al., 2009). It might also be of interest in the context of colocalized ITP and TK in the *Drosophila* ipc-1 neurons to note that in the silk moth *Bombyx* a receptor was identified that can be activated both by TK and a splice form of ITP (Nagai-Okatani et al., 2016).

Finally, there is a pair of large and widely arborizing *interneurons* in the lateral brain that produce at least three neuropeptides: AstA, MIP, and natalisin (Diesner et al., 2018) (**Figure 2A**). These cells are designated ICLI (Jiang et al., 2013). The peptides coexpressed in the ICLI neurons have been assigned individual functions in earlier studies, as listed next; the question is how they might act together to orchestrate a behavior or physiological function when coreleased from these neurons. The only known role of natalisin so far is in fecundity (Jiang et al., 2013), whereas the other peptides display several functions. MIP regulates mating, satiety and sleep stabilization (Oh et al., 2014; Min et al., 2016; Jang et al., 2017) and AstA is known to regulate feeding and sleep (Hergarden et al., 2012; Hentze et al., 2015; Chen et al., 2016b). These actions are probably via interneuronal pathways, some of which may converge on IPCs and insulin signaling. It would be of interest to search for further roles of natalisin since at this point it is not clear how this peptide could cooperate with AstA and MIP. Also, to grasp the compound function of the three peptides produced by the ICLI neurons it would be interesting to genetically interfere with activity of these cells. Do they regulate food ingestion and postprandial sleep, or some other behavior?

A systematic screen of peptidergic neurons in the larval brain of *Drosophila* revealed many of the same cases of coexpression seen in adults and added a few more (Park et al., 2008). The additional combinations in neurosecretory cells are dromyosuppressin (DMS) and ITP, and in other neurons of the brain DMS/sNPF, TK/MIP, NPF/DH31, and DSK/AstC (**Figure 2B**). A recent study also indicated that some of the important Hugin cells of the SEG, known to be at the interface between gustatory inputs and regulation of feeding in larvae, not only express hugin-PK, but also are likely to be cholinergic (Schlegel et al., 2016). The cell groups displaying this coexpression are the hugin-PC and hugin-VNC/PH cells.

### Coexisting Peptides in Neuroendocrine Cells of the Ventral Nerve Cord

Neurosecretory cells of the VNC regulate some functions overlapping those of the brain, but also have several distinct roles (Nässel and Winther, 2010). In the VNC cells colocalized peptide hormones also appear to coordinate several targets to orchestrate physiology and behavior. Some peptidergic interneurons or efferents discussed below utilize co-expressed neuropeptides and SMNs in various ways such as feedback facilitation of input neurons or signaling to reproductive organs.

In the thoracic neuromeres there is a set of six large FMRFamide-expressing neurons (Tv1-3) with release sites in a plexus of axon terminations in the dorsal neural sheath of the VNC (Lundquist and Nässel, 1990; Schneider et al., 1993). In abdominal neuromeres there are segmental neurons producing different peptide hormones, such as Capa peptides, CCAP, Bursicon, LK, DH44, orcokinins, and the glycoproteins GPA2/GPB5, (Cantera and Nässel, 1992; Luan et al., 2006; Sellami et al., 2011; Chen et al., 2015; Zandawala et al., 2018) (**Figures 3A,B**). Posteriorly in abdominal ganglia there are peptidergic neurosecretory cells (or efferent neurons) that supply axon terminations to the posterior intestine. These produce PDF, ITP, AstA, proctolin, and DILP7 (Anderson et al., 1988; Nässel et al., 1993; Yoon and Stay, 1995; Kean et al., 2002; Dircksen et al., 2008; Miguel-Aliaga et al., 2008; Cognigni et al., 2011; Sellami et al., 2011).

Neurons of the abdominal ganglia display several cases of coexpressed peptides (**Figures 3A,B**). In *Drosophila* larvae there are seven pairs of segmental neurosecretory cells in the abdominal ganglia (A1–A7) that express LK, designated ABLKs (Cantera and Nässel, 1992; de Haro et al., 2010) (**Figure 3B**). In adults the number of ABLKs is 20–22, by addition of 4–6 larger cells anteriorly in the abdominal ganglion (Luo et al., 2013; Alvarez-Rivero et al., 2017) (**Figure 3A**). LK is one of several diuretic hormones in *Drosophila* (Terhzaz et al., 1999) and is likely to be released into the circulation from the ABLKs that have axon terminations along the abdominal body wall and heart. LK acts on stellate cells of the Malpighian tubules to increase fluid secretion across the epithelium (Terhzaz et al., 1999; Halberg et al., 2015). Recently it was found that the ABLKs also express another diuretic peptide, the corticotropin-releasing factor-like DH44 (Zandawala et al., 2018) (**Figures 3A,B**). DH44 acts on another cell type in Malpighian tubules, the principal cells, also to stimulate fluid secretion (Cabrero et al., 2002). Thus, it appears as if the ABLKs release LK and DH44, and that both regulate diuresis, but via different epithelial cell types and intracellular mechanisms [Cl^-^ transport and cAMP mediated transport, respectively (Cabrero et al., 2002, 2014)]. Furthermore, the effect of the two hormones on secretion is additive (Zandawala et al., 2018). Both LK and DH44 have been shown to also regulate survival during stress induced by desiccation (Liu et al., 2015; Cannell et al., 2016). By targeted knockdown of each peptide in ABLKs it was found that either DH44 or LK signaling from these cells is sufficient for the regulation of resistance to desiccation, as well as ionic and starvation stress (Zandawala et al., 2018). However, regulation of food intake was only affected by DH44 knockdown in ABLKs. It should be noted that these two peptides are produced, but not colocalized, also in other neurons of the CNS, and that in these neurons DH44 and LK may serve other functions related to the circadian clock, sleep, metabolism, and reproduction (Lee et al., 2015; Cavey et al., 2016; Murakami et al., 2016).

There is a set of segmentally distributed neuroendocrine cells along the midline of abdominal ganglia that express DILP7 both in larval and adult abdominal ganglia (Miguel-Aliaga et al., 2008; Castellanos et al., 2013) (**Figures 3B,C**). In the most anterior pair, the interneurons DP1 with axons ascending to the brain, DILP7 is colocalized with sNPF and Cha expression (Nässel et al., 2008) (**Figure 3B**). It was found that the larval DP1 neurons are part of a circuit that integrates nociceptive (mechanosensory) inputs utilized in an escape response (Hu et al., 2017). In this circuit the DP1 neurons signal with sNPF back to their input neurons, specific nociceptive sensory neurons, and DILP7 is not necessary for the integrative function of the circuit. Probably acetylcholine is also not involved in this response since blocking regular neurotransmission has no effect on the response (Hu et al., 2017).

The posterior DILP7 neurons are more heterogeneous, especially in adult flies. Some are interneurons, others are efferents with axons to the hindgut or reproductive organs; additionally some neurons are adult-specific and sexually dimorphic (Castellanos et al., 2013) as shown in **Figure 3C**. The embryo-derived cells express only DILP7 in both sexes, whereas the neurons specific to the adult in males produce DILP7, serotonin and glutamate and innervate the seminal vesicle, but are not required for fertility (Castellanos et al., 2013). The female post-embryonic cells only express DILP7 and glutamate, and appear to be motor neurons that innervate the oviduct and are required for fertility.

Another case of sex-specific expression of a neuropeptide and a coexpressed neurotransmitter was demonstrated in the abdominal ganglia of adult flies where males have a set of Crz producing interneurons that also express Cha (Tayler et al., 2012). These neurons act via efferent Crz-receptor expressing serotonergic neurons that innervate the male accessory glands where they regulate sperm transfer. The role of acetylcholine in the signal transfer to the serotonergic neurons was not investigated.

In summary of the above section, one can infer that colocalized peptide hormones may orchestrate organismal physiology by acting on relevant targets, and that neuropeptides can provide feedback signals to presynaptic neurons. However, the functional role of coexpressed neuropeptides and SMNs remains to be explored in VNC interneurons and efferents.

## Proctolin as a Cotransmitter of Glutamate in Motor Neurons of the Ventral Nerve Cord

When the neuropeptide proctolin was first mapped to neurons in insects it was noted that in the cockroach *Periplaneta americana* certain slow skeletal motor neurons produce this peptide in addition to glutamate (Adams and O’Shea, 1983). Neuron stimulation or depolarization by potassium application triggered proctolin release onto the target muscle, a coxal depressor. A cotransmitter role was suggested since proctolin produces a delayed and sustained muscle contraction without actually depolarizing the muscle (Adams and O’Shea, 1983). This response differed from the normal response of muscle to activation of the Ds motor neurons, which is a transient depolarization and rapid contraction typical of glutamate stimulation (Adams and O’Shea, 1983).

Also in *Drosophila* certain motor neurons express proctolin (Anderson et al., 1988; Taylor et al., 2004). In *Drosophila* larvae especially the segmental mid-line motor neurons designated RP2 coexpress glutamate and proctolin (Luo et al., 2017) (**Figure 3D**). Application of proctolin onto body wall muscle in *Drosophila* larvae indicated muscle fiber specific actions and induced dose dependent slow contractions (Ormerod et al., 2016). Proctolin also potentiated nerve-evoked muscle contractions. Knockdown of proctolin receptor decreased thermal preference and larval crawling at higher temperature (Ormerod et al., 2016).

## Colocalized Neuropeptides in Abdominal Neurons Coordinate Ecdysis Motor Behavior

**FIGURE 4:**
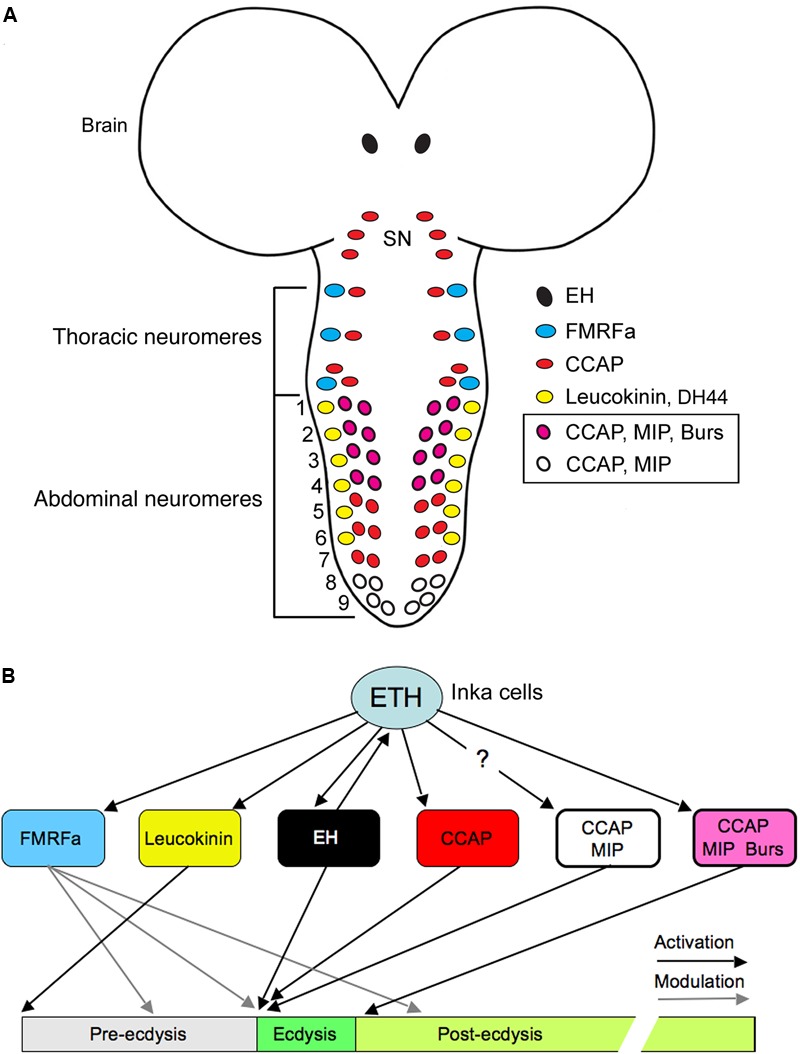
Neurons and neuropeptides that control ecdysis motor activity in *Drosophila*. **(A)** Schematic depiction of peptidergic neurons in the larval CNS that express the ecdysis triggering hormone receptor ETHR-A. These neurons respond to ETH in a sequence and trigger ecdysis behavior as shown in **(B)**. The color-coding depicts the expression of various neuropeptides in the neurons (note that some colocalize 2–3 peptides). ETH is released from peritracheal Inka cells (not shown). SN, subesophageal neuromeres 1–3; ETH, ecdysis triggering hormone; FMRFa, FMRFamide; EH, eclosion hormone; CCAP, crustacean cardioactive peptide; MIP myoinhibitory peptide; burs, bursicon. Redrawn from Kim et al. (2006), Nässel and Winther (2010).

In larval *Drosophila* there are sets of neuroendocrine cells in abdominal ganglia that regulate motor activity during molting behavior (**Figure 4**). These neurons express colocalized CCAP and bursicon and a subset produces also a third peptide, MIP (Kim et al., 2006; Luan et al., 2006). As seen in **Figure 4**, these neurons together with other abdominal and thoracic neurons that use CCAP, FMRFamide or LK express receptors for ecdysis triggering hormone (ETH) and when activated by ETH they signal in a temporal sequence (with feedback inhibition) to coordinate the ecdysis motor behavior (Kim et al., 2006, 2015). It is not yet clear how the co-localized MIP and CCAP act together in generating the ecdysis motor pattern, but bursicon released from some of the CCAP neurons is likely to act on other targets that regulate post-ecdysis phenomena (Luan et al., 2006). Also the role of DH44 coexpressed in the LK producing ABLKs (Zandawala et al., 2018) is not known. Since LK recently was found to play a hormonal role in regulating fluid transport in trachea during larval molts (Kim et al., 2018), it is possible that coreleased DH44 acts in a similar fashion.

## Coexpressed Neuropeptides and Neurotransmitters in the Clock Neurons of the Brain

**FIGURE 5:**
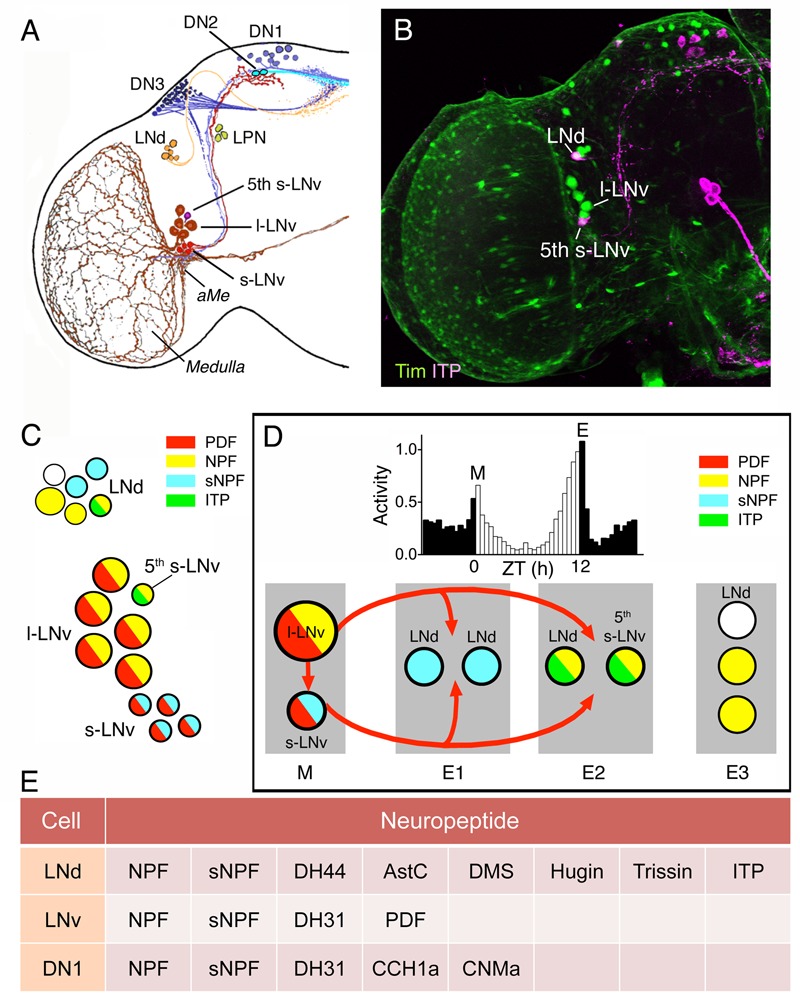
Several neuropeptides are colocalized in different types of clock neurons in *Drosophila*. **(A)** The different types of clock neurons in one brain hemisphere. There are lateral ventral neurons (s-LNv and l-LNv), lateral dorsal neurons (LNd), dorsal neurons (DN1–DN3), and a set of lateral posterior neurons (LPN). aMe, accessory medulla. This panel is originally from Helfrich-Förster et al. (2007), but slightly modified in Johard et al. (2009). **(B)** Expression of Tim-Gal4-driven GFP (green) and immunolabeling for ion transport peptide (ITP; magenta). From Johard et al. (2009). **(C)** Neuropeptides in LNd and LNv neurons are colocalized in different patterns. The figure is compiled from data in Johard et al. (2009) and Schlichting et al. (2016). Note that the s-LNvs also produce the amino acid transmitter glycine (Frenkel et al., 2017) and l- LNvs the cytokine Upd1 (Beshel et al., 2017). **(D)** The clock output generates daily activity with peaks in the morning (M) and evening (E) and low activity at night and mid-day. This activity is generated by clock neurons that act as morning (M) and evening (E1–E3) oscillators. These express different combinations of neuropeptides and some interactions between LNvs and other neurons are known to be by means of pigment-dispersing factor (PDF) shown in red arrows. The roles of other peptides are less known so far (but, see **Figure 6**). This figure is compiled in part from data in (Schlichting et al., 2016). **(E)** This table shows additional neuropeptides in clock neurons indicated by singe cell transcriptomics. Note that the cell types listed were assayed as groups and there is no information on peptides in subtypes of neurons in these groups. Thus it is not known to what extent the neuropeptide transcripts are coexpressed in specific neurons; this is especially prominent for the relatively large group of DN1 neurons, which may be heterogeneous. Data mined from Abruzzi et al. (2017).

Daily activity and physiology of animals is synchronized with the 24 h cycle of earth’s rotation around its axis with the aid of an endogenous circadian clock. In *Drosophila* the master clock is situated in the brain and consists of about 150 neurons in 8 bilateral groups (Nitabach and Taghert, 2008; Schlichting et al., 2016) shown in **Figures 5A,B**. The first neuropeptide to be associated with clock neurons was PDF, expressed by small and large lateral ventral neurons, s-LN_v_s and l-LN_v_s (Helfrich-Förster, 1995; Renn et al., 1999). In addition to PDF, several other neuropeptides have been mapped to different clock neurons in *Drosophila* (Johard et al., 2009; Hermann et al., 2012; Kunst et al., 2014; Schlichting et al., 2016; Abruzzi et al., 2017). In several sets of clock neurons different combinations of these neuropeptides are colocalized (**Figures 5C–E** and **Table 2**). As will be shown below, most studies of clock neuron peptides have analyzed their functions one by one, but in some cases it is evident that colocalized peptides target different neurons of the clock network, and it has also been shown that PDF and an unidentified SMN act together on a common set of target neurons (Choi et al., 2012).

The two sets of main pacemaker neurons (morning and evening oscillators) the l-LN_v_s/s-LN_v_s and LN_d_s display different combinations of the neuropeptides PDF, NPF, sNPF, and ITP (**Figures 5C,D**) and some subsets of neurons also produce SMNs such as acetylcholine, and glycine (**Table 2**). The l-LN_v_s are known to express PDF and NPF (at least in some cells), as well as the cytokine unpaired 1 (Upd1), and the 5th s-LN_v_ produces ITP, NPF and acetylcholine (Johard et al., 2009; Schlichting et al., 2016; Beshel et al., 2017). Single cell transcriptomics identified further neuropeptide candidates in LNds: DH44, AstC, DMS, Hugin peptides, and trissin (Abruzzi et al., 2017) (**Figure 5E**). The dorsal neurons (DNs) are located in three clusters DN1-3 and are diverse in terms of neuroactive substances. The two DN1a (anterior) neurons produce glutamate, IPNamide (from NPLP1 precursor) and diuretic hormone 31 (DH31) (Shafer et al., 2006; Hamasaka et al., 2007; Goda et al., 2016). The other DN1 neuron cluster located more posteriorly (DN1p) as a group expresses transcripts for DH31, NPF, sNPF, CCHamide1, and CNMamide (Abruzzi et al., 2017) (**Figure 5E**). This peptide transcript expression was determined by RNA sequencing of dissociated DN1p neurons, and it is therefore not clear in which specific DN1p neurons they are located or to what extent the neuropeptides are colocalized. However, DH31 was mapped to some DN1p neurons by immunolabeling (Kunst et al., 2014).

Of all these peptides PDF is the one extensively investigated for distinct roles in the clock network and as an output of LN_v_s (see Nitabach and Taghert, 2008; Taghert and Nitabach, 2012; Schlichting et al., 2016). Also sNPF and NPF play defined roles within the network, but for the other peptides the main information available is on network outputs monitored as activity, sleep or other behaviors. With the exception of colocalized sNPF and PDF, discussed below, there are no clear data on the outcome of cotransmission by neurons in the clock circuit. However, to provide an idea of what the individual neuropeptides/SMNs do, and how one could approach analysis of cotransmission, I briefly summarize the known signaling roles of these substances in the clock network.

**FIGURE 6:**
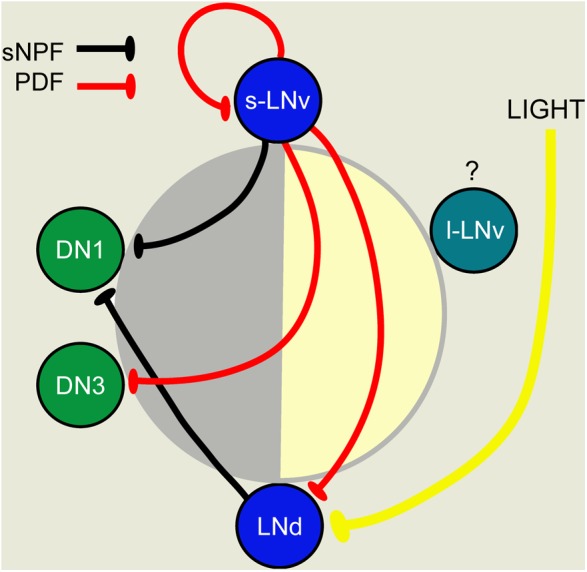
A scheme depicting PDF-, sNPF-, and light-mediated interactions that orchestrate sequential Ca^2+^ activity phases in different pacemaker groups. Each pacemaker group is represented by one neuron. The position of cells in the day-night circle (yellow–gray) indicates the peak phase of Ca^2+^. Both PDF and sNPF signals inhibit the target neurons and suppress these from being active when the sender neurons (s-LNv for PDF; s-LNV and LNd for sNPF) are active. Light cycles act together with PDF to delay Ca^2+^ phases in LNds. This figure was redrawn from Liang et al. (2017).

As seen in **Figure 5D**, PDF from both small and large LN_v_s act on sets of clock neurons that generate evening activity (evening oscillators) and the large LN_v_s signal to small ones. The l-LN_v_s receive light inputs both from the compound eyes and the extraretinal photoreceptors of the eyelet, whereas the s-LN_v_s only from the latter (Schlichting et al., 2016). All groups of neurons in the network shown in **Figure 5D** (M, E1, and E2 oscillators), except l-LN_v_s, express the PDF receptor (PDFR), and s-LN_v_s receive light information from large cells, but also seem to utilize the PDFR as autoreceptors (see Taghert and Nitabach, 2012). More specifically, these autoreceptors inhibit s-LN_v_ activity and PDF release and thereby play a role in setting the phase of daily outputs, including locomotor activity (Liang et al., 2017). The PDF signaling to other clock neurons is inhibitory and causes delays in calcium activity in follower neurons, LNd and DN3 (see **Figure 6**).

Some other neuropeptides, such as sNPF and NPF, found in clock neurons also act in the clock network in patterning daily rhythms. Release of sNPF from s-LN_v_s and LNds acts to sculpt the DN1 activity period at night by suppressing DN1 activity at other times (Liang et al., 2017). The s-LN_v_s receive negative PDF feedback in an autocrine loop, and both sNPF and PDF suppress Ca^2+^ levels in other pacemakers (**Figure 6**), thereby providing a neuropeptide-mediated chain of sequential inhibition and delay in the network that ensures phase-setting of neuronal activity (pacemaker entrainment) (Liang et al., 2017). This interplay between sNPF and PDF in a defined network is also a good example how a single neuron type (s-LN_v_) can use sNPF and PDF to target different neurons (**Figure 6**). Another example of division of labor between sNPF and PDF released from s-LN_v_s will be discussed below in the context of a developmental transition.

NPF was found in 1–3 of the l-LN_v_s and in the 5th s-LN_v_ and function was analyzed in flies with the NPF expressing clock neurons genetically ablated (Hermann et al., 2012). Such ablation eliminates the neurons with both NPF and other colocalized substances and results in flies with prolonged free-running period in constant darkness, an advanced phase of the evening activity peak and reduced amplitude of this peak. Further experiments suggested that this phenotype arose from ablation of the NPF expressing LNds and the 5th s-LN_v_ (Hermann et al., 2012). Diminishing NPF by RNAi in clock neurons only had a minor effect and slightly advanced the evening activity phase. With simultaneous knockdown of both PDF and NPF the effect was stronger and resembled that seen after ablation of the neurons. However, the specific role of NPF in the clock circuit needs to be further characterized by future experiments, and especially the combined roles of the neuropeptides, known to be colocalized with NPF, require further study.

Some neuropeptide roles in the clock neurons involve aspects other than signaling within the network to generate rhythmic activity patterns. ITP release from the 5-th s-LN_v_s is under clock control and knockdown of ITP in these cells and LNds results in reduced evening activity of the flies and an increase in night activity (Hermann-Luibl et al., 2014). These authors also demonstrated that interference with ITP did not affect PERIOD (PER) cycling, suggesting that the peptide is part of an output pathway rather than being a signal within the clock network. Knockdown of both ITP and PDF resulted in hyperactive flies that were arrhythmic in constant darkness, and also displayed reduced sleep both during mid-day and night (Hermann-Luibl et al., 2014).

Neuropeptide F in clock neurons regulate aspects of mating behavior (Lee et al., 2006; Hamasaka et al., 2010; Kim et al., 2013), sleep-wake behavior (Chung et al., 2017), and indirectly the peptide regulates circadian gene expression in the fat body (Erion et al., 2016). Again, it is not known whether combined actions of co-expressed peptides affect these behaviors. Another peptide, sNPF, has been implicated in regulation of sleep: this peptide in s-LN_v_s is promoting sleep without affecting feeding (Shang et al., 2013). DH31 in the clock system was demonstrated as a wake-promoting neuropeptide acting before dawn (Kunst et al., 2014). Finally, DH31, and to a lesser extent PDF, acting on DN2 neurons regulate night time temperature preferences in flies and PDF mainly regulates locomotor activity rhythm (Goda et al., 2016). Interestingly, these authors propose that DH31 acts via the somewhat promiscuous PDF receptor in DN2 neurons to decrease temperature preference at night onset.

Another aspect of clock outputs is regulation of developmental transitions such as shedding the old cuticle (ecdysis) in insects. Timing of molts relies on production of the steroid hormone ecdysone in the prothoracic gland and is regulated by PTTH produced in two pairs of LNCs (Mc Brayer et al., 2007). A recent study revealed that timing of the final molt, the adult emergence from the puparium (eclosion), is regulated by the s-LN_v_s signaling with sNPF, but not PDF, to the PTTH neurons (Selcho et al., 2017). This s-LN_v_ signal thereby serves to coordinate the central clock with the local one in the prothoracic gland (Selcho et al., 2017). Of interest in this review: this is a clear example of a distinct separation of functions of two colocalized neuropeptides.

What about the SMNs in the clock neurons? DN1s were shown to promote sleep by glutamate release that inhibits pacemaker neurons (both morning and evening oscillators) and a feed-back circuit ensures generation of the mid-day siesta and night sleep, especially in males (Guo et al., 2016). The inhibitory neurotransmitter glycine in LN_v_s contributes to synchronization of the circadian network (Frenkel et al., 2017). These authors showed that diminishing the glycine production in LN_v_s increases the period length, without affecting the locomotor activity rhythm of the flies. Thus, fast inhibitory neurotransmission in addition to PDF plays a role in synchronizing the clock circuit, and it was proposed that PDF and glycine released from s-LN_v_s might signal to the LNds to affect the period of activity (Frenkel et al., 2017). An earlier study provided evidence that activation of PDF autoreceptors on s-LN_v_s modulates release of PDF and a non-identified SMN resulting in a rhythm acceleration and increased morning activity (Choi et al., 2012). The SMN mediating this light-induced phase-shift might be glycine.

Do signal molecules from clock neurons act on circuits outside the *bona fide* clock network to regulate behavior that is not directly involving locomotor activity or sleep? A recent finding was that the leptin-like cytokine Upd1 (Unpaired 1) is produced by LNv clock neurons (Beshel et al., 2017). The Upd1 receptor Domeless (Dome) is expressed in several NPF neurons in the brain, known to be orexigenic, and disruption of Upd1 signaling leads to increased food attraction and food ingestion as well as weight increase. These findings suggest that clock neuron-derived Upd1 suppresses NPF neuron activity and thereby food intake. It is not clear from the study whether NPF expressing clock neurons are among the ones expressing the Upd1 receptor Dome, or if the effect is on NPF neurons outside the principal clock circuit.

**FIGURE 7:**
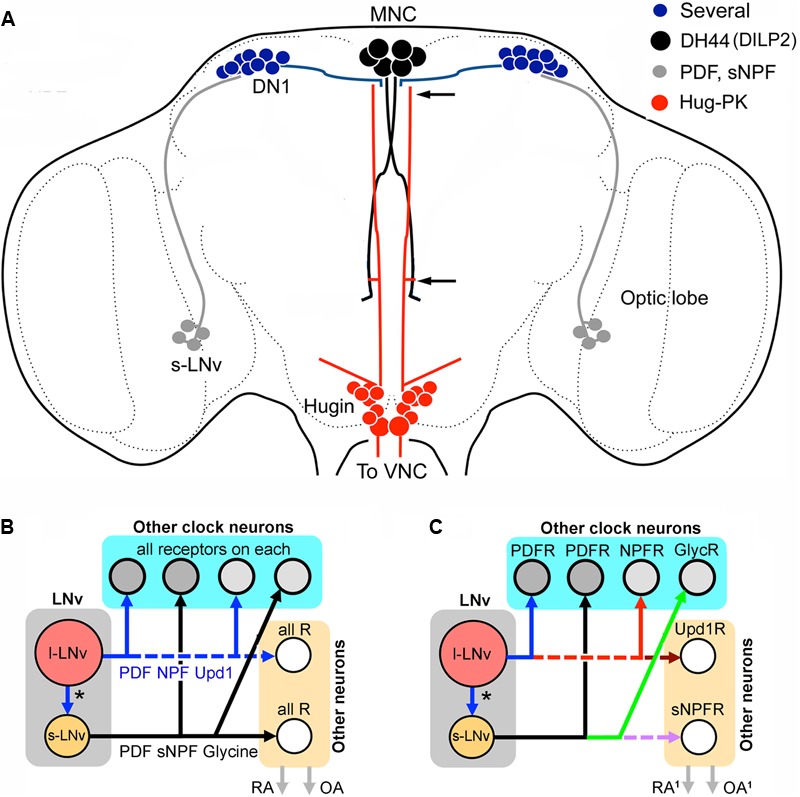
Networks of peptide cotransmission in the clock circuits of *Drosophila*. **(A)** Neuronal circuit that regulates locomotor activity proposed in King et al. (2017). It was suggested that s-LN_v_s signal to DN1s that in turn act on DH44-producing MNCs. These signal with DH44 to hugin cells in the SEG via the DH44-R1. Some of the hugin cells have axons that terminate in the VNC where they contact glutamatergic neurons in motor centers that generate locomotion. The two arrows indicate areas where interactions between MNCs and hugin cells can occur. It is not known which peptide/neurotransmitter of the s-LNvs and DN1s that signal in this pathway. For s-LNvs it could be PDF, sNPF or glycine and for DN1s there are several candidate peptides (see **Figure 5E**). Note that the MNCs also produce DILP2 (Ohhara et al., 2018). **(B)** The colocalized neuropeptides and other signaling molecules in the LN_v_s may target different constellations of neurons within and outside the clock circuitry; these are represented by “other clock neurons” and “other neurons,” respectively. The “other neurons” are effectors downstream to the clock circuits that regulate, e.g., rhythmic activity in behavior and physiology (RA), or neuronal systems that influence other behaviors such as reproduction, foraging and feeding (and indirectly metabolism), or produce systemic responses via hormone release (OA). In the simplest model, shown in **(B)**, all the target neurons for the large and small LN_v_s express receptors for all of the signal molecules released. Thus, targets of s-LN_v_s within and outside the clock circuit would all express receptors (R) for PDF, sNPF and glycine, and targets of l-LN_v_s have receptors for PDF, NPF and Upd1 (unpaired-1). Each of the neurons would thus receive multiple complementary signals. **(C)** Hypothetically the target neurons of the LN_v_s could be more diverse and express only subsets of the relevant receptors in different combinations. In this extreme example each target neuron type expresses only one receptor type and the downstream circuits could generate neuropeptide and signal substance-specific activity. Here, activity in, e.g., s-LN_v_s would simultaneously generate a more diverse set of actions within and outside the network. These could include some actions that are targeted only to neurons outside the bona fide clock network (e.g., the Upd1 or sNPF actions in this example). The outputs, RA^1^ and OA^1^, could be more diversified.

In summary of the above, it is known that in addition to locomotor activity and sleep, some of the outputs of the clock regulate specific behaviors such as feeding and reproduction. A few specific outputs of the clock are relevant to consider in the light of the coexpressed neuropeptides in the system. One pathway that was recently discovered consists of connections between clock neurons and output neurons regulating locomotor activity, without affecting feeding rhythm (King et al., 2017). This pathway, shown in **Figure 7A**, comprises connections from s-LN_v_ neurons to DN1s, that signal to DH44 neurons in the PI, which in turn connect to Hugin neurons in the SEG that via descending axons regulate glutamatergic premotor neurons in the VNC (Cavanaugh et al., 2014; King et al., 2017). These connections were established both by GRASP technique (GFP reconstitution across synaptic partners) and genetic manipulations. In this pathway it is postulated (but not clearly shown) that s-LN_v_s signal with PDF to the DN1 neurons, which in turn use an unknown substance to activate the DH44-MNCs. The signal between the MNCs and Hugin neurons was shown to be DH44, presumably via its receptor DH44-R1 and these communicate with glutamatergic neurons in motor circuits possibly with the peptide hug-PK (King et al., 2017). This pathway has some room for additional or alternative signals from s-LN_v_s (sNPF or glycine) and for DN1s there are several candidate peptides (see **Figure 5E**). It can be mentioned that also brain insulin producing cells (IPCs) are under modulation by DN1s giving rise to rhythmic action potential firing frequency in IPCs (Barber et al., 2016). This study suggests that IPCs, although they have cell autonomous nutritional inputs that also affect the firing rhythm, are under additional circuit regulation. Thus, IPC signaling that affects feeding and metabolism is under rhythmic clock control (Barber et al., 2016). It is not known which of the multiple substances in DN1s that modulate IPC activity.

Finally, it has been demonstrated that there is a link between the central clock and the peripheral clock in the fat body in *Drosophila*. Many gene transcripts cycle in the fat body, but some cytochrome P450 transcripts cycle independently of the fat body clock and are instead dependent on NPF expressing brain clock neurons, probably LNds (Erion et al., 2016). It is not clear how the signal from the NPF clock neurons reaches the fat body, but it is likely to be via interactions with neurosecretory cells such as IPCs or other MNCs. It is also not entirely clear whether NPF is the only required signal from these clock neurons since NPF knockdown was less effective than silencing the NPF neurons (Erion et al., 2016). As shown above the NPF expressing LN_v_s also produce ITP or PDF (see **Figure 5C**) and the LNds perhaps produce even further neuropeptides (**Figure 5E**).

Clearly, cotransmission plays a fundamental role in different parts of the clock circuitry and is of key importance for understanding the organization and logic of the regulatory hierarchy in the network. How does the clock network use multiple SMNs and neuropeptides for internal and external signaling? An attempt to summarize hypothetical cotransmission outputs from LNv clock neurons is shown in **Figures 7B,C**. In **Figure 7B** the simplest scheme assumes that all direct target neurons of each small or large LN_v_ express all the receptors for the released peptides, inferring that each target within the clock network, or outside, would be modulated by several substances. The scheme in **Figure 7C** displays the other extreme where each of the substances acts on different target neurons (with a corresponding receptor), thus producing divergent outputs that can generate specific effects in different parts of the network or outside. The outside network action is shown for example by DN1s interacting with IPCs and DH44 expressing MNCs. Another molecule that might target non-circuit neurons is Upd1 that may signal to orexigenic NPF non-clock neurons. This scheme would enable single clock neuron types to modulate both network properties and activity related to other behaviors such as feeding, metabolism and reproduction. Probably neither of the two schemes is fully correct. Even a scheme that is a hybrid of the two proposed ones would probably be subject to an additional possibility: the presence of given receptors may not predict signaling outcomes. The action of specific ligands on receptors in different cell types may result in responses that differ depending on cell type and context.

## Cotransmission in the Olfactory System

Subpopulations of each of the components of the olfactory system, the olfactory sensory neurons (OSNs), local interneurons (LNs), and projection neurons (PNs) that carry sensory signals to higher brain centers, have all been shown to display colocalization of different combinations of neuropeptides and SMNs. Studies of olfactory sensory processing and odor-guided behavior have explored a few cases of cotransmission of neuropeptides and SMNs to reveal mechanisms of presynaptic facilitation or inhibition that regulates state-dependent food search, as discussed below.

### Olfactory Sensory Neurons Colocalize Acetylcholine and sNPF or MIP

**FIGURE 8:**
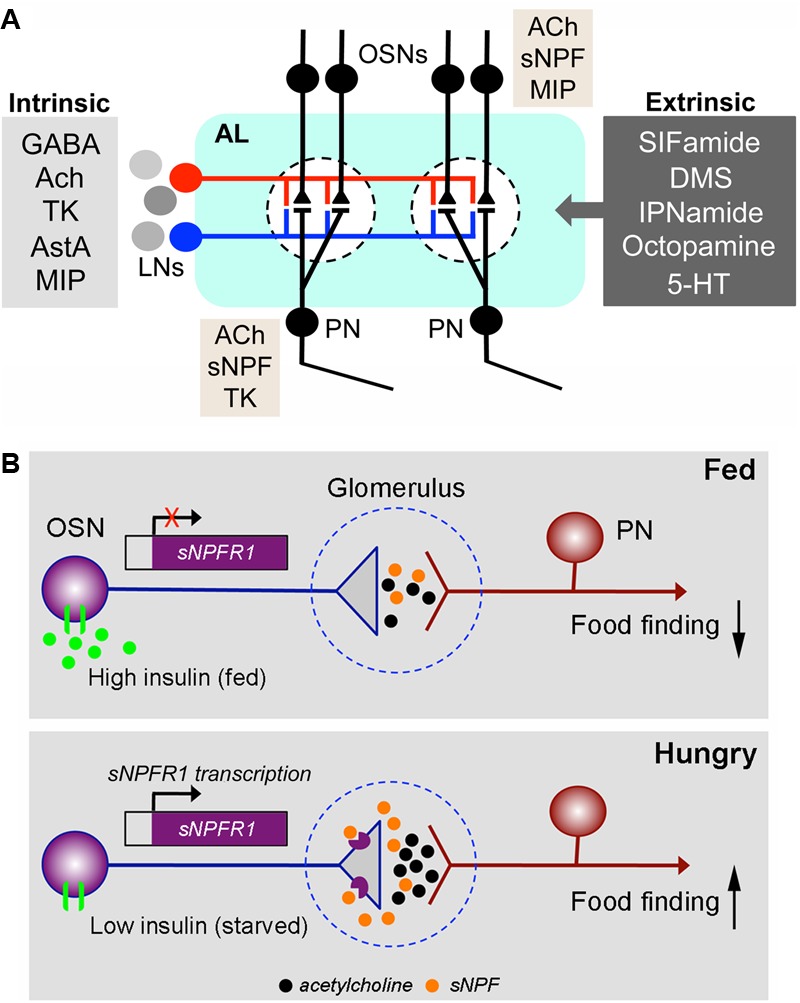
Coexpression and neuromodulation in the olfactory system. **(A)** Neuromodulation in the *Drosophila* antennal lobe. The antennal lobe (AL) is shown highly schematically with only two glomeruli (dashed outlines). Inputs to the glomeruli are from OSNs in the antenna and labial palps. The OSNs synapse on PN that relay signals to higher brain centers (MBs and lateral horn). The OSNs and PNs are modulated by local neurons (LNs), which form intrinsic modulatory circuits and by extrinsic neurons that utilize several neurotransmitters/neuromodulators. The LNs are either GABAergic, cholinergic (Ach), or in some cases glutamatergic. The former two types are known to colocalize the neuropeptides tachykinin (TK), allatostatin-A (AstA) or myoinhibitory peptide (MIP) (Carlsson et al., 2010), whereas it is not known whether glutamatergic ones colocalize any peptide. The extrinsic neurons utilize SIFamide, dromyosuppressin (DMS), IPNamide (from precursor NPLP1), octopamine or serotonin (5-HT) (Dacks et al., 2006; Sinakevitch and Strausfeld, 2006; Roy et al., 2007; Carlsson et al., 2010). It is not known whether any of these extrinsic neurons colocalize other neurotransmitters/neuropeptides. Additionally, a subpopulation of the OSNs coexpress Ach and sNPF (Nässel et al., 2008; Carlsson et al., 2010) and in females some OSNs with Ir-type receptors coexpress MIP (Hussain et al., 2016a). Recent reports from single cell transcriptomics suggest that some PNs may express sNPF and others TK in addition to Ach (Croset et al., 2017). The general outline of this figure is redrawn from (Lizbinski and Dacks, 2017). **(B)** The OSNs are regulated presynaptically by insulin and sNPF. This figure, based on a paper by Root et al. (2011), shows an OSN synapsing on a PN in the antennal lobe of a fed *Drosophila* fly (top) and a hungry one (bottom). The synapse is located within a glomerulus. In the fed fly, the level of circulating insulin is high and the activated insulin receptor (dInR) on the OSN inhibits transcription of the sNPF receptor, sNPFR1. Thus, there is low expression of the sNPFR1 presynaptically on the OSN axon termination and signal transfer with acetylcholine at the synapse is weak. As a result, food search/finding is low. In the hungry (starved) fly, insulin levels are low and the transcription of sNPFR1 in the OSNs is activated. Consequently, presynaptic sNPFR1 expression increases and released sNPF activates the presynapse leading to enhanced release of acetylcholine and thus increased signaling in the synapse: food search/finding increases. This is an example of an autocrine presynaptic regulation. The figure is slightly altered from Nässel (2012), which was compiled from Root et al. (2011).

Sensory cells, including OSNs, in *Drosophila* utilize acetylcholine as their primary neurotransmitter (Buchner et al., 1986; Yasuyama and Salvaterra, 1999; Masse et al., 2009). A scheme of the neurons and SMNs and neuropeptides in the olfactory system is shown in **Figure 8A**.

It was found that a subset of the OSNs of the antennae and maxillary palps coexpress sNPF (Nässel et al., 2008). The sNPF expressing OSNs supply axon terminations to at least 13 of the approximately 50 glomeruli of the antennal lobe (Carlsson et al., 2010). Each glomerulus receives sensory input from one specific odor channel (olfactory receptor type) (Couto et al., 2005; Fishilevich and Vosshall, 2005) and, thus, a subset of these odor signals can be relayed with acetylcholine and modulated by an intrinsic neuromodulator, such as sNPF.

A specific role of sNPF in modulation of food odor detection was demonstrated in *Drosophila* (Root et al., 2011). A hungry fly displays vigorous food search (foraging behavior) and obviously pays more attention to food-related odors. This odor sensitivity is regulated by systemic insulin signaling, and an autocrine loop in the OSNs involving sNPF and its receptor sNPFR (**Figure 8B**). The insulin receptor (dInR) is expressed on OSNs and so are both sNPF and its receptor, sNPF-R (Root et al., 2011). In hungry flies circulating insulin (DILP) levels are low and expression of sNPF-R is high in OSNs and food odor stimulation triggers release of sNPF, which via action on the autoreceptor increases release of acetylcholine, the primary transmitter at the synapse with PNs. This potentiates the odor signal to higher brain centers and leads to increased food search (Root et al., 2011). After feeding the DILP levels increase in the circulation and activation of the dInR in OSNs in antennae causes an inhibition of transcription of the sNPFR and thus minimal autocrine sNPF signaling leading to decreased activation of PNs and decreased food search (Root et al., 2011). Thus, sNPF is a cotransmitter of acetylcholine that facilitates synaptic activation dependent on insulin signaling over an extended period until the fly has found and ingested food.

Another neuropeptide expressed in a small subpopulation of OSNs is MIP, and also the MIP/sex peptide receptor is expressed in the same cells (Hussain et al., 2016a,b). This is seen in female flies in OSNs expressing Ir41a/Ir76b ionotropic receptors that are sensitive to polyamines. Similar to sNPF the MIP peptide acts in an autocrine loop in OSNs to regulate polyamine attraction in mated flies, and sex peptide does not seem to be involved (Hussain et al., 2016a,b).

### Local Interneurons of the Antennal Lobe Colocalize GABA and Tachykinin and Several Other Combinations

The LNs of the antennal lobe use acetylcholine, GABA and glutamate as neurotransmitters (Masse et al., 2009; Seki et al., 2010). As seen in **Table 2** there are LNs coexpressing different combinations of SMNs and neuropeptides: some GABAergic LNs produce TK (Ignell et al., 2009), some cholinergic LNs express MIP or AstA, furthermore TK is coexpressed with MIP or AstA, and MIP was found together with AstA in LNs (Carlsson et al., 2010).

The role of TK signaling has been analyzed in the olfactory system. TK from LNs acts on TK receptors (DTKR, *TkR99D*) by suppressing calcium and synaptic transmission in the OSNs, thereby providing presynaptic inhibitory feedback (Ignell et al., 2009). It was later shown that also TK receptor expression in OSNs (with Or42b and Or85a receptors) is regulated by feeding-dependent DILP signaling (Ko et al., 2015). The coordinated action of the two peptides decreases synaptic outputs from Or42b OSNs (positive valence) and increases Or85a OSNs (negative valence) simultaneously, thereby diminishing the overall attractive value of food odors. During starvation, reduced insulin levels leads to upregulation of sNPFR and DTKR in their respective OSNs resulting in an increased attraction of food odors (Ko et al., 2015). Whereas sNPF facilitates cholinergic transmission in OSNs, it is not clear whether TK acts to modulate GABA transmission in LNs.

## Another Case of Presynaptic Facilitation: Acetylcholine and sNPF in Kenyon Cells of the Mushroom Bodies

The MBs are prominent paired neuropils in the protocerebrum of insects (see **Figure 1**) and known to be centers of olfactory learning and memory (Heisenberg, 2003). The MBs are formed by thousands of intrinsic neurons called Kenyon cells and various types of extrinsic neurons of efferent and afferent nature (Takemura et al., 2017). In *Drosophila* the MBs have been under intense investigation for many years (see Heisenberg, 2003; Davis, 2005; Perisse et al., 2013), but only recently was a SMN assigned to the Kenyon cells. Evidence was put forth for the production of acetylcholine by these cells and that this neurotransmitter mediates the output of the MB via nicotinic receptors on MB output neurons, MBONs, and is critical for learning (Barnstedt et al., 2016). It had been shown earlier that a major subpopulation of the Kenyon cells express sNPF (Johard et al., 2008), and a neuromodulator role of sNPF was indicated in a study of sugar reward olfactory memory (Knapek et al., 2013). Now it is known that sNPF potentiates the response to acetylcholine in MBONs suggesting that the peptide presynaptically facilitates the response to the fast neurotransmitter (Barnstedt et al., 2016) as shown earlier in the OSNs (Root et al., 2011). Since food-associated memory formation is enhanced by hunger (Krashes et al., 2009) it would be of interest to determine whether sNPF signaling in the MBs is regulated by the nutritional state of the fly, as was shown in the OSNs (Root et al., 2011).

## Colocalization of Peptides in Intestinal Endocrine Cells

Like the neurosecretory cells of the brain and VNC, the midgut endocrine cells in some cases coexpress peptide hormones that might act on different targets to orchestrate physiology and maintain homeostasis. However, functional aspects of coreleased hormones have not been investigated so far.

**FIGURE 9:**
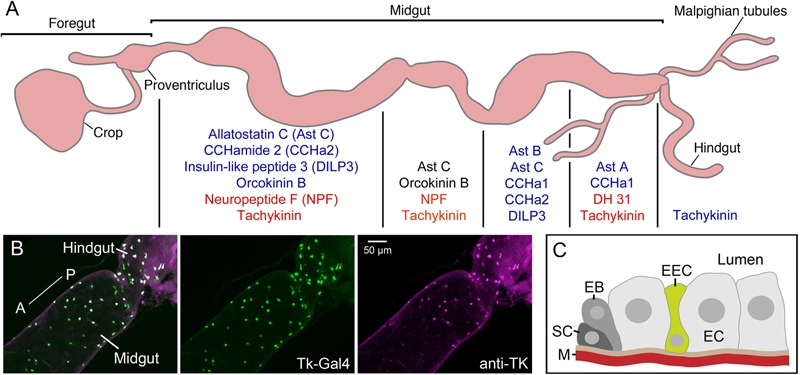
Neuropeptides and peptide hormones in the *Drosophila* intestine. **(A)** In the midgut enteroendocrine cells (EECs) produce neuropeptides/peptide hormones in a region-specific manner. Four midgut regions are shown here [based on (Veenstra and Ida, 2014)]. Peptides produced are shown in blue text, and those that are colocalized in EECs in the different regions are shown in red or black text (NPF and tachykinin, AstC and orcokinin B, DH31 and tachykinin). DH31 is calcitonin-like diuretic hormone 31. There are also EECs producing tachykinin in the anterior hindgut. This figure is compiled from Veenstra et al. (2008); Veenstra and Ida (2014). **(B)** EECs in the posterior midgut and anterior hindgut produce tachykinin (TK) as demonstrated by Tk-Gal4 driven GFP (green) and antiserum to TK (magenta). From Liu et al. (2016b). **(C)** Schematic depiction of an EEC located between enterocytes (EC). The EECs may sense nutrients in the gut lumen and release peptide either locally in a paracrine fashion or into the circulation outside the muscle layer (M). The other cells shown are stem cells (SC) and enteroblasts (EB) that are responsible for renewal of the gut epithelium. This figure was redrawn from a figure in Lemaitre and Miguel-Aliaga (2013).

In the *Drosophila* midgut epithelium there are different types of endocrine cells, known as enteroendocrine cells, EECs (**Figure 9**). In adult flies 10 different neuropeptides/peptide hormones have been detected in such EECs (Veenstra et al., 2008; Veenstra and Ida, 2014) (see **Figure 9**). Additionally DILP3 is produced by intestinal muscle cells (Veenstra et al., 2008) and sparse bursicon expression was seen in some EECs (Scopelliti et al., 2014). In EECs of the anterior and middle midgut TK and NPF are colocalized, in the midportion orcokinin B and allatostatin C, and in the posterior end TK and DH31 (**Figure 9A**) (Veenstra et al., 2008; Veenstra and Ida, 2014; Chen et al., 2016a). Of note is that in the larval gut anteriorly located MIP producing EECs were shown to also express Cha, suggesting that they produce acetylcholine, which may act in paracrine signaling (LaJeunesse et al., 2010). Little is known about the function of gut-derived peptides in *Drosophila*. Data suggests that the EECs can release peptide hormones into the circulation, as well as use them for paracrine signaling (Winther and Nässel, 2001; Veenstra et al., 2008; Reiher et al., 2011; Li et al., 2013; Song et al., 2014; Wegener and Veenstra, 2015; Chen et al., 2016b; Liu and Jin, 2017).

It was demonstrated *in vitro* that intestines of locust and cockroach display depolarization-induced release of TK and that hemolymph levels of TK increased after starvation, suggesting that EECs can release peptide into the open circulation (Winther and Nässel, 2001). Other indirect evidence also supports the possibility of hormonal action of EEC-derived peptides (AstA and CCHamide2) on distant targets, such as the brain (Li et al., 2013; Chen et al., 2016b). One study has shown that TK from EECs acts in a paracrine fashion in the midgut of *Drosophila* to regulate local lipid production in the enterocytes, thereby contributing to lipid homeostasis systemically (Song et al., 2014). These authors also showed that EEC-derived TK does not affect behavior of the fly, indicating that the gut peptide does not affect neurons in the CNS. Possible roles of peptides colocalized with TK were not investigated in the same context. Further local functions of gut peptides have been suggested for other insects: modulation of gut peristalsis, secretion of digestive enzymes and regulation of ion transport (see LaJeunesse et al., 2010; Nässel and Winther, 2010; Reiher et al., 2011). It is noteworthy that some EECs (NPF- and DH31-producing) express gustatory receptors, such as for instance Gr43a, known to be a fructose sensor (Park et al., 2016). Thus, nutrient sensing could be cell autonomous in EECs and regulate peptide release.

To search for sites of action of EEC-derived gut peptides one can start by screening for expression of their cognate receptors in various tissues. Using available transcriptome databases Veenstra et al. (2008), Veenstra and Ida (2014) identified transcripts of receptors of all gut peptides except orcokinin B in the midgut, and AstA-R2 also in the hindgut. Most of these receptors are also expressed in the CNS, suggesting that the gut peptides theoretically could act in both these tissues. Many other targets of gut peptides are possible, since inter-organ communication appears to be important aspect of maintaining homeostasis in the fly (Owusu-Ansah and Perrimon, 2015; Liu and Jin, 2017).

## Concluding Remarks and Future Perspectives

This review highlighted some neuronal and endocrine systems in the CNS and intestine of *Drosophila* where neuroactive substances are coexpressed. These include neurosecretory cells in the brain and VNC, chemosensory cells and interneurons of the olfactory system, nociceptive neurons, intrinsic neurons of the mushroom body, different clock neurons, as well as EECs of the intestine. However, in spite of these reports on cellular co-distribution of neuropeptides/neuropeptides and neuropeptides/SMNs in *Drosophila*, analysis of the functional relevance of cotransmission has lagged far behind. Furthermore, reports published so far on mapping of coexpression to specific neurons are likely to cover only a small fraction of the actual cases. This is suggestive from a few recent reports analyzing transcriptomes of single dissociated cells in the *Drosophila* brain where preliminary data already uncovered plenty of new combinations of colocalized substances (Abruzzi et al., 2017; Croset et al., 2017; Davie et al., 2017) shown in **Tables 3, 4**. These novel data constitute a rich source for future systematic mapping of neuropeptides and SMNs to neurons and circuits in the brain of *Drosophila* by imaging techniques.

Several features of neuronal cotransmission mentioned in the introduction remain unexplored in *Drosophila*. For instance, it is not clear to what extent peptidergic neurons in general are connected to other neurons by traditional synapses and to what degree peptide signaling is paracrine (or parasynaptic). However, a recent analysis of early *Drosophila* larvae revealed synaptic connections between some identified sets of peptidergic neurons, including the hugin cells and IPCs (Schlegel et al., 2016), but evidence for volume transmission remains to be provided. Related to this, it is not known how far neuromodulators can diffuse within the insect CNS. In general these questions are more acute in *Drosophila* than in mammals since almost no mapping of neuropeptide/SMN receptors is available for insects and thus the spatial match between release sites and receptors is not known. Another aspect that remains to be investigated in *Drosophila* is the complex dynamics of cotransmission in modulation of network activity, matching that available for the stomatogastric ganglion in crustaceans (see Nusbaum et al., 2001, 2017; Marder and Bucher, 2007; Marder, 2012; Nusbaum and Blitz, 2012). Most *Drosophila* studies employ genetic tools to tamper with signaling components, and even when combined with dynamic calcium imaging these manipulations are commonly too crude to reveal relevant dynamic changes in the network properties or neuronal pathways. However, *Drosophila* has proven excellent for analysis of neuromodulation in single synapses in the olfactory system (Root et al., 2008; Ko et al., 2015). In the following I will discuss the few advances made in cotransmission in *Drosophila* and highlight some of the areas where further studies would be valuable.

### Cotransmission Analyzed in *Drosophila*

**Table 5** summarizes neurons and circuits where analysis of cotransmission has been employed in *Drosophila*. This Table also highlights that sNPF plays multiple roles in synaptic facilitation or other cotransmission in a multitude of circuits in the CNS, which is in line with its widespread distribution in large numbers of neurons of different types (Nässel et al., 2008). For instance in sets of mushroom body Kenyon cells and antennal OSNs synapsing in the antennal lobes it has been shown that acetylcholine and sNPF are colocalized and that the neuropeptide acts presynaptically to potentiate signaling with the SMN (Root et al., 2011; Barnstedt et al., 2016). Possibly this is a common role of this peptide in various neurons co-expressing SMNs and sNPF described earlier (Nässel et al., 2008) (see **Tables 2, 4**). It would be of interest to test whether sNPF also acts presynaptically to regulate release of colocalized neuropeptides for instance in clock neurons or even neurosecretory cells. In the s-LN_v_ clock neurons, which colocalize PDF and sNPF, it has been demonstrated that these two peptides have distinct functions and target neurons. Thus, sNPF, but not PDF from s-LN_v_s target the PTTH-producing neurosecretory cells in the brain to coordinate the central clock with that in the prothoracic gland and thereby time the ecdysone production and adult eclosion (Selcho et al., 2017). Another case is where s-LN_v_s target DN1 clock neurons with sNPF, whereas they act on LNds, DN3s and themselves with PDF in a circuit that ensures phase shifts in the activity of clock neurons, as part of clock entrainment (Liang et al., 2017). So far it is not known whether sNPF and PDF from s-LN_v_s also act together on any target neurons. A final example of sNPF action is in feedback regulation of the presynaptic sensory neurons by a set of DILP7/sNPF-expressing interneurons in a nociceptive pathway (Hu et al., 2017).

**Table 5:** Summary of established functional roles of colocalized neuropeptides/SMNs in *Drosophila*.

Neuron type^1^	Peptide/SMN^2^	Cotransmission/divergent roles	Reference
MB Kenyon cells	sNPF, ACh	Presynaptic facilitation (learning)	Barnstedt et al., 2016
OSNs (sensory)	sNPF, ACh	Presynaptic facilitation (olfaction)	Root et al., 2011
OSNs (sensory)	MIP, ACh	Presynaptic facilitation (olfaction)^3^	Hussain et al., 2016a
DP1 (interneuron)	sNPF, DILP7	Feedback facilitation (nociception)^4^	Hu et al., 2017
s-LNv (clock)	sNPF, PDF^5^	Different targets (activity phase shift)	Liang et al., 2017
s-LNv (clock)	sNPF, PDF^5,6^	Different targets (Ecdyson production)	Selcho et al., 2017
DLP (neurosecretory)	sNPF, Crz^5^	Different targets (CNS and systemic)	Kubrak et al., 2016
Ipc-1 (neurosecretory)	sNPF, TK^5^	Different targets (CNS and systemic)	Kahsai et al., 2010
IPC (neurosecretory)	DILPs, DSK^5^	Different targets (systemic)	Nässel and Vanden Broeck, 2016
ABLK (neurosecretory)	LK, DH44^5^	Different targets (systemic)	Zandawala et al., 2018

*^*1*^In adult CNS except DP neurons which are in larvae. ^*2*^Only the ones studied are listed. ^*3*^In mated females. ^*4*^DP neurons signal back to mechanosensory cells with sNPF, DILP7 plays no role in this feedback circuit. ^*5*^These neurons/neuroendocrine cells use the colocalized peptides to target different neurons or tissues for different actions, to orchestrate function. ^*6*^During final molt (adult eclosion).*

Neurosecretory cells producing two or more peptide hormones that have been studied so far seem to orchestrate physiology by sharing some target cells/tissues, but also appear to have some unique targets of the individual peptides. For instance IPCs of the brain produce four DILPs and two DSKs (**Figure 2A**) and these seem to be transcriptionally regulated individually and probably their release is also differentially controlled [summarized in (Nässel and Vanden Broeck, 2016)]. More importantly the individual DILPs of the IPCs appear to have distinct physiological roles (with some redundancy), although they share a single receptor tyrosine kinase, dInR (see Brogiolo et al., 2001; Grönke et al., 2010; Nässel and Vanden Broeck, 2016). Furthermore, there are two DSK receptors (GPCRs) and these cholecystokinin-like peptides have multiple functions [summarized in (Nässel and Winther, 2010; Nässel and Williams, 2014)]. In summary, the combined roles of DILPs and DSKs from IPCs seem to be to orchestrate satiety and post feeding physiology (Söderberg et al., 2012).

Another example is a set of LNCs that produce the peptides Crz, sNPF, and proctolin (**Figure 2A**). Experimental data suggest that sNPF regulates DILP signaling from the IPCs in the brain and thereby affects stress responses and metabolism (Kapan et al., 2012), whereas Crz appears to act primarily as a hormone in a systemic fashion to target Crz receptors in the fat body to regulate responses to metabolic stress (Kubrak et al., 2016). This indicates that the same set of LNCs act on different targets in the CNS and periphery to orchestrate a systemic response.

A final example is a set of neurosecretory cells, ABLKs (**Figure 3A**), in abdominal ganglia that were shown to target Malpighian tubules with the diuretic hormones DH44 and LK to regulate secretion and stress responses, but also have other targets that affect feeding, and water retention in differential ways for the two peptides (Zandawala et al., 2018).

### Cotransmission in *Drosophila* in the Future

Clearly, cotransmission in *Drosophila* is an open field of mostly uncharted territory. There is no clear choice of a general study system, since there are many different aspects of cotransmission to be investigated and each aspect may be best studied in a specific circuit or neuroendocrine system. Thus, I list a few systems as examples where advances could be made.

For analysis of cotransmission and roles of multiple neuromodulators in regulating network properties, as in the stomatogastric ganglion in crustaceans (see Nusbaum et al., 2017), there is unfortunately no easily accessible simple motor network in flies in which electrophysiological recordings accompanied by application of neuroactive substances and drugs could reveal dynamics of network modulation. However, progress is being made in larval *Drosophila* in a neuronal network regulating feeding and locomotion that is accessible to electrophysiology and optogenetics (Schoofs et al., 2014; Hückesfeld et al., 2015). This network includes the peptidergic Hugin cells of the SEG whose extensive connectome was described recently (Schlegel et al., 2016).

The anatomically well-described olfactory system is amenable to calcium imaging, electrophysiology, and application of neuroactive substances while stimulating the OSNs with odorants (Masse et al., 2009; Seki et al., 2010; Wilson, 2013) and is already utilized in analysis of cotransmission at the first synapse between OSNs and PNs (Root et al., 2008; Ko et al., 2015). There are many types of LNs in the antennal lobe, known to colocalize neuropeptides and SMNs and the concerted actions of for instance GABA and TK or acetylcholine and MIP in shaping olfactory information passing on to higher centers remain a future challenge. Also the novel discovery that LNs may signal with both acetylcholine and two different neuropeptides, sNPF and TK (Croset et al., 2017) adds another layer to plasticity in olfactory signaling to the MBs and lateral horn worthy of investigation. Similarly, the mechanisms behind sNPF facilitation of acetylcholine transmission in mushroom circuitry (Barnstedt et al., 2016), especially possible nutrient-state dependence during olfactory learning, merits further study.

Clearly the clock circuitry is another promising system for analysis of the function of colocalized neuropeptides and SMNs due to the wealth of data on network properties and roles of individual neuron groups. Existing data suggests extensive involvement of neuropeptides in different parts of the network (Johard et al., 2009; Schlichting et al., 2016; Liang et al., 2017), whereas the role of SMNs is relatively uncharted and cotransmission with the two remains to be investigated. Another interesting possibility is that various clock neurons utilize their colocalized signaling substances to target different follower neurons. This has already been indicated for the s-LN_v_s that signal with sNPF and PDF to different neurons of the circuit (Liang et al., 2017). Likewise it is suggestive, but not proven, that Upd1 from l-LN_v_s, that also colocalize PDF and NPF, target non-clock neurons (Beshel et al., 2017). The genetic manipulations usually performed are relatively crude, even when using temporarily controlled manipulations, and may miss subtle dynamics of cotransmission. Thus, there is a need to combine with analysis of synaptic signaling and neuromodulation, such as performed in the crustacean stomatogastric system.

Finally, the complex endocrinology of *Drosophila* with extensive interorgan communication to orchestrate behavior, maintain homeostasis, or regulate developmental processes remains an important research field with much promise for the future (Rajan and Perrimon, 2011; Owusu-Ansah and Perrimon, 2015). Neurosecretory cells are relatively easy to access for analysis of hormonal roles of multiple peptides (see Söderberg et al., 2012; Zandawala et al., 2018). It is feasible to knock down colocalized neuropeptides or their receptors in target tissues, individually or several together, and analyze systemic effects on the organism.

In conclusion analysis of cotransmission in *Drosophila* is still in its infancy and hopefully this review will convince the reader that *Drosophila* is a promising model organism to employ for future functional studies of colocalized neuroactive substances.

## Author Contributions

DN conceived and wrote this review.

## Conflict of Interest Statement

The author declares that the research was conducted in the absence of any commercial or financial relationships that could be construed as a potential conflict of interest.
